# Monitoring trace minerals and heavy metals in liver of free-living large herbivores in the Netherlands

**DOI:** 10.3389/fvets.2026.1751586

**Published:** 2026-02-24

**Authors:** Inês Marcelino, Gustavo Monti, Perry Cornelissen, Evelyn Bassingthwaighte, Jasper het Lam, Deon van der Merwe, Wim H. M. van der Poel

**Affiliations:** 1 Infectious Disease Epidemiology, Wageningen University and Research, Wageningen, Netherlands; 2 Department Nature and Society, Staatsbosbeheer, Lelystad, Netherlands; 3 Institute for Biodiversity and Ecosystem Dynamics, University of Amsterdam, Amsterdam, Netherlands; 4 School of Veterinary Science, University of Queensland, Gatton, QLD, Australia; 5 Department of Ruminant Health, Royal GD, Deventer, Netherlands; 6 Department of Physiological Sciences, College of Veterinary Medicine, Oklahoma State University, Stillwater, OK, United States; 7 Department of Research and Development, Royal GD, Deventer, Netherlands; 8 Department of Virology and Molecular Biology, Wageningen Bioveterinary Research, Lelystad, Netherlands

**Keywords:** heavy metals, Heck cattle, Konik horses, nature reserve, red deer, reference intervals, trace minerals

## Abstract

Trace minerals are essential for animal health but can also, together with heavy metals, have a negative impact, making their monitoring crucial to assess animal health. These elements were examined through a long-term post-mortem monitoring system based on routine liver sampling for Heck cattle, Konik horses and red deer in place at the Oostvaardersplassen nature reserve in the Netherlands, using data from this system to determine reference intervals and investigate trends in liver trace element concentrations. Throughout the monitoring programme, inductively coupled plasma mass spectrometry was used to measure concentrations of trace minerals and heavy metals, including arsenic, cadmium, chromium, cobalt, copper, iron, lead, manganese, molybdenum, nickel, selenium, vanadium, and zinc. Species-specific patterns in trace element profiles were identified, with red deer showing comparatively higher copper levels and horses elevated iron and lead levels. Temporal declines in certain elements, including iron and lead, were observed across all species. Seasonal and age-related variations were also evident. Importantly, reference intervals estimated in this study differed from livestock standards, in particular for copper and selenium, highlighting the need for species- and context-specific reference intervals when assessing health in free-living herbivores. These findings provide valuable baseline data for ongoing environmental and health monitoring in minimally managed, multi-species populations at the reserve, highlighting the importance of mineral surveillance in free-living animals to enhance wildlife health assessment, track long-term environmental changes, and support management decisions in nature reserves across the Netherlands and more globally.

## 1 Introduction

Trace elements are fundamental to animal health, influencing several physiological functions and serving as important indicators for health monitoring. These elements can be classified as essential or non-essential. Essential elements are required to maintain balanced physiological functions, contributing to growth, immune function, and reproduction, with examples including copper (Cu), selenium (Se), and zinc (Zn). Imbalances in their concentrations can negatively affect animal health, either through deficiencies caused by low levels or toxicity from increased exposure (1, 2). In contrast, non-essential elements, such as arsenic (As), cadmium (Cd) and lead (Pb), do not have a physiological function. However, these elements can accumulate in animal tissues through environmental exposure, often associated with anthropogenic contamination, and in some cases adversely impact animal health, for example causing nephrotoxicity and neurological effects (2). Therefore, monitoring both types of elements is important for assessing the health of individual animals and populations.

Determining mineral concentrations is a classical approach to assess animal health, typically in a veterinary context, as reference intervals (RIs) provide essential, species-specific benchmarks. These intervals offer a valuable frame of reference for interpreting laboratory results by representing the range of values typically found in healthy animals (3, 4). By differentiating normal mineral levels from potential imbalances (i.e., deficiency or excess), RIs support the evaluation of animal health and inform decisions related to diagnosis, nutritional management, and the assessment of environmental exposure.

Trace element measurements are commonly performed in various tissues, including blood, hair, muscle, and liver. In particular, the liver is considered a suitable tissue for these measurements, as it is a major organ for the storage and deposition of mineral elements (1, 2, 5). In livestock, mineral concentrations and their RIs are generally well defined, facilitating straightforward interpretation of mineral status as deficient, adequate, high, or potentially toxic (2, 6). Conversely, while such RIs are documented and standardized for livestock, their establishment and validation in free-living and wild animals are less consistent and require further systematic evaluation and improvement (7).

Wild herbivores such as different deer species and European bison (*Bison bonasus*), as well as domestic species such as cattle and horses, are often introduced into nature areas as part of their management, contributing to the goals of nature restoration and conservation (8). As these animals are introduced in often small and isolated areas, monitoring their wellbeing is essential. Mineral concentration measurements provide a valuable tool for health assessment. However, applying livestock RIs to free-living herbivores presents challenges due to differences in management and ecology. Unlike livestock, which are primarily managed to achieve economic production goals, free-living herbivores have different purposes and management conditions. These animals typically have continuous access to natural grazing grounds year-round, rely on natural water sources, and usually do not receive supplementary feed, factors that affect mineral intake and likely result in mineral concentrations different from those in livestock and wild herbivores kept under managed conditions (e.g., farmed or captive populations) (9–12). Moreover, livestock RIs often reflect productivity-related demands, such as growth, reproduction, and lactation (2), while the mineral status of free-living herbivores is primarily influenced by natural environmental availability, forage composition, and physiological maintenance needs (13, 14). Thus, direct application of livestock RIs to free-living herbivores may lead to inadequate conclusions, highlighting the need for specific RIs for wild and free-ranging herbivores to ensure accurate monitoring and informed management.

In the Netherlands, a health monitoring programme has been in place in the *Oostvaardersplassen* (OVP) nature area since the late 1990s. Within this programme, trace element concentrations are measured in the livers of Heck cattle (*Bos taurus*), Konik horses (*Equus caballus*) and red deer (*Cervus elaphus*). Livestock RIs are currently employed to assess the health status of these large herbivores. Given this context, the aims of this study were to determine RIs for 13 trace minerals and heavy metals in the large herbivore populations at the OVP, and to investigate potential variation in the levels of these elements between animal species, time, and other biological factors at the nature reserve. To achieve this, livers from Heck cattle, Konik horses, and red deer from the OVP were collected during post-mortem examination and then analyzed to measure trace element concentrations. By investigating trace element concentrations in large herbivores livers in a nature reserve area, this study contributes with insights on health monitoring of large herbivores in nature reserves in the Netherlands.

## 2 Materials and methods

### 2.1 Study area and animal population

This study was carried out at the nature reserve *Oostvaardersplassen* (OVP), which is part of the National Park *Nieuw Land* in the Netherlands. Three large herbivore species were introduced in the area between the 1980s and 1990s: Heck cattle (*Bos taurus*), Konik horses (*Equus caballus*), and red deer (*Cervus elaphus*). These herbivores graze the area to create a diverse landscape for wetland related birds. The animals were introduced from different countries in Europe, and prior to introduction all animals received health checks according to import regulations (15).

The OVP is a eutrophic wetland of 55 km^2^, consisting of marshland (36 km^2^) and a dry border zone (19 km^2^). The marshland is mainly covered by shallow water bodies and large reed beds with patches of willow shrubs and trees. The dry border zone primarily consists of inundated and non-inundated short grazed homogeneous grasslands. The inundated grasslands are flooded from December until April. The area is part of a polder which was established in 1968 and located in a former inland saltwater lake that was changed into a freshwater lake in 1932. Thus, the soil at the OVP consists of nutrient and mineral rich marine clay.

Historically, large herbivore populations at the OVP were primarily regulated by food availability, winter severity, and density-dependent competition for food. During periods of high food availability and relatively mild winters, population sizes increased substantially, resulting in increased grazing pressure and reduced diversity in vegetation structure (16). As a result, herbivore shelter in the border zone was reduced, and the animals were given access to surrounding forest areas, which consist of planted shrubs and trees combined with patches of grassland, tall herbs (thistles, stinging nettle) and reed vegetation. Red deer use the entire area of the nature reserve including the marshland and all the forest areas available to them, while Konik horses and Heck cattle do not use the marshland, primarily inhabit the grassland areas, and can also use a smaller forest area. The whole nature area is fenced, and no large predators are present.

In 2018, a policy was introduced to promote biodiversity and enhance animal welfare (17). This policy focuses on managing herbivore populations through active culling to reduce grazing pressure. Apart from culling for population control, injured animals are removed for welfare reasons, and supplementary feeding of cattle and horses is carried out since 2018 when the average Heck cattle population body condition score is below two (scale from one to five). The policy aims to balance ecosystem management with animal welfare, particularly emphasizing the physical health and social behavior of large herbivores.

### 2.2 Sample collection and processing

Sample collection for this study was conducted through an ongoing health monitoring system established at the OVP in 1997 to assess population health, independent of population control management. The system involves regular inspections conducted jointly by rangers of the State Forestry Department [*Staatsbosbeheer* (SBB)] and a veterinarian. Through the system, animals are submitted for pathological examination and subsequent liver sampling, with blood and fecal samples also collected for pathogen testing [though the latter is outside the scope of this study and has been reported in a previous study (15)]. The number of animals sampled was determined by pathogen detection and statistically calculated based on population size and design prevalence, assuming 100% test sensitivity and specificity (18). For a herd of 300 to 500 animals, detecting one or more infected animals with a design prevalence of 25% or higher required a minimum of 11 animals per species (cattle, red deer, or horses) annually. This led to an annual target of at least 33 necropsies per year. Due to practical constraints, this target could not be met consistently each year. Over the study period (2003–2023), a total of 346 animals underwent post-mortem examination and trace element concentration measurements, comprising 167 Heck cattle, 96 red deer, and 83 Konik horses. OVP rangers collected these samples throughout the year, aiming for simple random sampling whenever possible. However, when random sampling was not feasible, non-random (purposive and convenience) sampling was adopted. Shot animals were transported to the Dutch Animal Health Services (Royal GD) facilities where pathological examination was performed by eight board certified veterinary pathologists according to standard protocols. Liver sampling and subsequent analyses were conducted using previously published methods (19, 20), and were performed in an ISO 17025 compliant laboratory. During the post-mortem examination, a minimum of 100 g of liver was collected, homogenized and stored at −20 °C until further analysis. After homogenisation, liver samples were dried for 4 h at 103 °C to determine dry weight (dw). Following the drying process, approximately 1 g of liver tissue was used for analysis. The samples were digested in 6 ml of 65% nitric acid in a microwave oven digestion system (200 °C, 4 min ramp, 5 min hold, maximum pressure 40 MPa, medium stirring). Following digestion, samples were diluted with 25 ml ultrapure water and with added internal standards (germanium, scandium and thallium; NIST-traceable, Inorganic Ventures). Element concentrations were measured using Inductively Coupled Plasma Mass Spectrometry (ICP-MS; Agilent) for the following elements: arsenic (As), cadmium (Cd), chromium (Cr), cobalt (Co), copper (Cu), iron (Fe), lead (Pb), manganese (Mn), molybdenum (Mo), nickel (Ni), selenium (Se), vanadium (V), and zinc (Zn); with quantification based on external calibration standards prepared from certified reference solutions (Inorganic Ventures). Trace element concentrations were expressed in mg/kg dw.

### 2.3 Data analysis

A retrospective cross-sectional study was conducted regarding trace element concentration data from cattle, red deer and horses, collected through the pathological and liver analyses obtained for the monitoring system at the OVP between the years of 2003 to 2023. Data handling and analyses were performed in R (version 4.4.1) (21), using RStudio (version 2024.04.2+764). Data manipulation used the *tidyverse* package (22), and data visualization was done using *ggplot2* (23).

Data exploration and statistical analyses were conducted on liver element concentrations, and associated variables: age, sex/pregnancy status, health status of sampled animals, body condition, year of sampling, season and annual average sward height of the grazed grasslands. Age was categorized as young (< 2 years old), adults (2–10 years old), and seniors (>10 years old). Sex and pregnancy were combined and classified as pregnant female, non-pregnant female, and male. Health status was determined using a decision flowchart (Supplementary Figure 1). To determine RIs according to the guidelines (4), the sampled animals were classified into two groups, either “indicative of disease” or “non-indicative of disease.” This classification was based on body condition, gross pathology/histology findings, and laboratory test results, including direct pathogen detection and serology. The full details of the classification are presented in the supporting information (Health status classification, Supplementary Figure 1). Body condition was grouped into not poor (including good, normal, moderate categories) and poor. Seasons were grouped into two categories: autumn-winter and spring-summer. Annual average sward height values for the grazed grasslands were obtained from long-term vegetation monitoring conducted along fixed transects (500–1,000 m) in the dry grasslands of the OVP, with height and cover of grasses and low herbs estimated every 50 m in a 2 × 2 m quadrat. Within each quadrat, average height of grasses and low herbs was estimated using a measuring stick with a division in centimeters, and cover was estimated visually as the percentage of the quadrat covered by these plants. The number of transects increased from 4 in 1990 to 14 in 2020, reflecting grassland expansion, ensuring consistent coverage of vegetation dynamics.

For statistical analyses, samples with concentrations below the limit of quantification (LOQ) were assigned a value equal to half the LOQ value for each element (Table 1). Elements for which more than 50% of the measured concentrations fell below the LOQ were excluded from the statistical analyses. For cattle and red deer, these elements were As, Cd, Cr, Ni, and V; for horses, As and Ni.

**Table 1:** Limit of quantification (LOQ) in mg/kg dw of each element as determined by inductively coupled plasma mass spectrometry (ICP-MS) in this study.

**Element**	**As**	**Cd**	**Cr**	**Co**	**Cu**	**Fe**	**Pb**	**Mn**	**Mo**	**Ni**	**Se**	**V**	**Zn**
LOQ	0.4	0.1	0.1	0.1	1	10	0.1	0.1	0.1	0.1	0.4	0.1	5

#### 2.3.1 Multivariate analysis

Redundancy analysis (RDA) was used as a multivariate approach to explore patterns of variation and similarity in trace element concentrations across cattle, red deer, and horses, and to assess how much of this variation could be explained by animal and environment variables. Specifically, RDA was applied to quantify the proportion of variation in animal trace element concentrations explained by species, demographic traits (age group, sex/pregnancy status), physiological condition (body condition, health status), temporal factors (year, season), and vegetation structure (average sward height per year). Prior to analysis, the distribution of trace element concentrations was evaluated, and consequently log-transformed to reduce skewness and minimize the influence of extreme values. The transformed data were then centered and scaled (z-transformed) to standardize variance across variables. Multicollinearity among explanatory variables was evaluated using Variance Inflation Factors (VIF) with the function *vif.cca* from the *vegan* package in R (24). RDA was implemented using the *rda* function from the same package. Statistical significance was assessed for the overall model, each variable (via marginal permutation tests), and canonical axes by permutation-based ANOVA (999 iterations) using the *anova.cca* function. RDA was performed on complete cases, restricting the analysis to samples collected between 2019 and 2023. Ordination results were visualized using *ggplot2* package (23). Convex hull polygons were used to represent species-specific group dispersion, and biplot vectors were used to indicate the contribution of individual trace elements to the ordination axes.

#### 2.3.2 Regression analysis

Regression analyses were conducted to quantify species and element specific associations between trace element concentrations and animal/environmental variables. Separate models were fitted for each trace element and animal species combination. Models included the predictors: year, age group, sex/pregnancy status, body condition, health status, season, and average sward height. To improve interpretability and meet model assumptions, linear models (LMs) with log-transformed trace element concentrations were used in most cases. Log transformation improved residual normality, stabilized variance, and reduced the influence of extreme values. Model assumptions were checked using diagnostic plots and statistical tests (e.g., Shapiro-Wilk for residual normality, Breusch-Pagan for homoscedasticity). In cases of mild/moderate assumption violations, log-transformed LMs were retained to preserve consistency across analyses. Collinearity was assessed using VIF values for fitted models. Model selection was guided by a combination of residual diagnostics, assumption checks, information criteria (AIC/BIC), and interpretability. Adjusted *R*^2^ values were included for each model to show how much of the variation in trace element concentrations is explained by the included predictors. Although a few trace element concentrations (e.g., Mo in cattle) were adequately modeled on the raw scale, log-linear models were generally preferred to ensure consistency and clarity across analyses. All regression models were performed using complete cases only. For categorical predictors, variables were excluded when they had only a single observed category among the complete cases.

#### 2.3.3 Correlation analysis

Spearman rank correlation coefficients were used to explore pairwise relationships among trace element concentrations within each species separately. Correlation analyses were intended to complement multivariate results. Correlations with coefficients *p* > 0.3 and *p* < 0.05 were considered potentially relevant and are presented in the Supplementary material.

#### 2.3.4 Reference intervals estimation

To estimate RIs, only the animals classified as “non-indicative of disease” were included (Heck cattle *n* = 38, red deer *n* = 57, Konik horses *n* = 53). Sample sizes differed between elements because some trace elements were analyzed throughout the full study period, whereas others were introduced later, resulting in element-specific variation in data availability. RIs and 90% confidence intervals (CIs) were calculated following the American Society for Veterinary Clinical Pathology (ASVCP) guidelines (4). Histograms and boxplots of trace element concentrations were visually inspected to identify potential outliers. Datapoints were rechecked in the original pathology report to verify that no processing error occurred. In the absence of documented error, these observations were retained in the primary RI estimation, as health status selection criteria was applied. Normality was assessed with both the Shapiro-Wilk and Anderson-Darling and the variable was considered normally distributed if both tests returned *p-value* > 0.05. Additionally, symmetry of the data was evaluated using Miao, Gel, and Gastwirth's test (R function *symmetry.test* from package *lawstat*) (25). Summary statistics were then computed.

RIs estimation methods were chosen based on sample size, data distribution and symmetry. Supplementary Figure 2 shows the decision flowchart used to select the most appropriate method based on the aforementioned guidelines. For sample sizes between 40 and 120 animals, the non-parametric method was used for non-normal distributions, and parametric method for normal distributions. For sample size between 20 and 40 animals, either the parametric or the robust methods were selected based on distribution, both before and after log transformation of data when needed. The CIs for the reference limits were either parametrically estimated (parametric method) or estimated with bootstrapping methods with 5,000 resamples (non-parametric and robust methods). Due to skewed distributions and the presence of tied values in the data, the percentile bootstrap method was used throughout (26). CIs that could not be reliably estimated due to statistical limitations, such as low sample size or limited data variability, were reported as Not Determined (ND). RIs and CIs were estimated using R function *refLimit* from package *referenceIntervals* (27). Further partitioning into subgroups such as age and sex was not explored due to insufficient sample size (*n* < 40) in at least one of the subgroups.

To evaluate the robustness of RI estimates to the influence of extreme values in the context of small to moderate sample sizes, an exploratory sensitivity analysis was performed. The same groups (“non-indicative of disease”) used for primary RI estimation were re-analyzed after identification of statistical outliers using Horn's algorithm, as recommended for RI studies in the presence of skewed distributions (4). Horn's algorithm applies a Box–Cox transformation, followed by detection of values outside Tukey's interquartile fences on the transformed scale. Values identified as statistical outliers were excluded only for the purpose of sensitivity analysis and were not considered indicative of analytical error or pathological status. RIs and 90% CIs were then re-estimated using the same methods as in the primary analysis. For each trace element, the number of excluded observations and changes in RI limits and CI widths were summarized and compared with the primary estimates to assess the influence of statistical outlier values on RI estimation. Horn's algorithm was applied using the *horn.outliers* function from the *referenceIntervals* R package (27).

## 3 Results

### 3.1 Description of study population

The number of samples collected and sampled population characteristics are shown in Table 2. Over the study period, most of the collected samples belonged to Heck cattle (48%), followed by red deer (28%) and Konik horses (24%). Within the cattle and red deer sample population, there were more females (>50%) sampled than males, while in horses the opposite occurred. The adult group had the highest number of samples (>~50%) across all three animal species. Regarding the health status of sampled animals, overall, 43% of sampled animals were classified as “non-indicative of disease,” while 57% were classified as “indicative of disease.” Within the “non-indicative of disease” group, the majority of animals had good or normal body condition (about 70% of each species), while a smaller proportion had moderate body condition (Supplementary Table 1).

**Table 2:** Characteristics and demographics of the sampled population.

**Categories**	**Heck cattle**	**Red deer**	**Konik horses**	**Total**
**Total**, ***n*** **(%)**^**a**^	167 (48.3)	96 (27.7)	83 (24.0)	346 (100)
**Age group**, ***n*** **(%)**				
Young (<2 years)	43 (25.7)	29 (30.2)	16 (19.3)	88 (25.4)
Adults (2–10 years)	82 (49.1)	45 (46.9)	52 (62.7)	179 (51.7)
Seniors (>10 years)	28 (16.8)	5 (5.2)	13 (15.7)	46 (13.3)
Unknown	14 (8.4)	17 (17.7)	2 (2.4)	33 (9.5)
**Sex/pregnancy**, ***n*** **(%)**				
Pregnant female	68 (40.7)	34 (35.4)	10 (12.0)	112 (32.4)
Non-pregnant female	45 (26.9)	28 (29.2)	26 (31.3)	99 (28.6)
Male	39 (23.4)	29 (30.2)	46 (55.4)	114 (32.9)
Unknown	15 (9.0)	5 (5.2)	1 (1.2)	21 (6.1)
**Body condition**, ***n*** **(%)**				
Not poor	84 (50.3)	70 (72.9)	65 (78.3)	219 (63.3)
Poor	64 (38.3)	19 (19.8)	16 (19.3)	99 (28.6)
Unknown	19 (11.4)	7 (7.3)	2 (2.4)	28 (8.1)
**Health status**, ***n*** **(%)**				
Non indicative of disease	38 (22.8)	57 (59.4)	53 (63.9)	148 (42.8)
Indicative of disease	129 (77.2)	39 (40.6)	30 (36.1)	198 (57.2)

^a^The sample numbers for each single mineral element varies.

### 3.2 Determinants of liver trace element concentrations

#### 3.2.1 Multivariate patterns (RDA)

**Figure 1:**
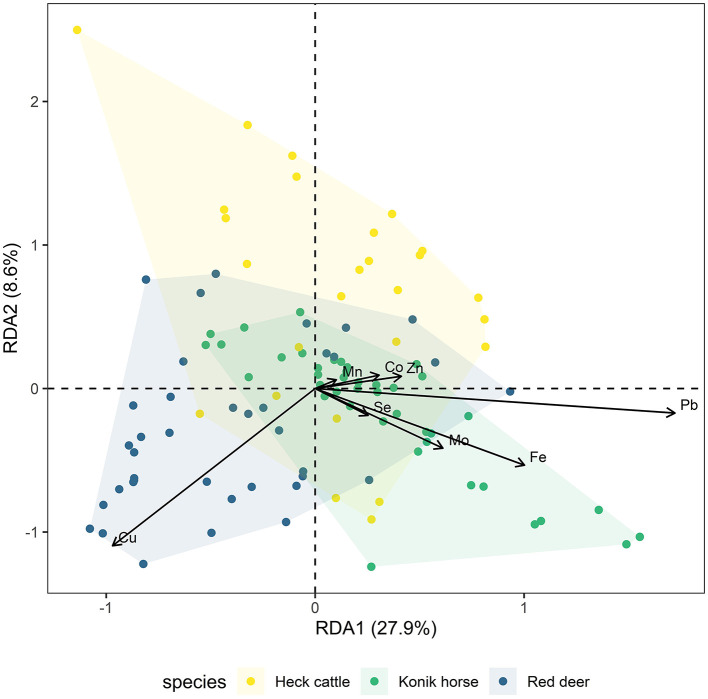
Redundancy Analysis (RDA) biplot based on mineral concentrations in Heck cattle (*n* = 26), Konik horses (*n* = 41), and red deer (*n* = 38) at the Oostvaardersplassen. Points represent individual samples, colored by species. Arrows indicate the direction and magnitude of mineral loadings. Axis labels include the percentage of variance explained by the corresponding dimension. Convex hull represent species-specific group dispersion.

Redundancy analysis (RDA) revealed clear multivariate patterns in liver trace element profiles across cattle, red deer, and horses, influenced by both species identity and biological and environmental factors (Figure 1, Supplementary Table 2). The constrained model explained 43.3% of the total variance in trace element concentrations (*p* = 0.001, adjusted *R*^2^ = 36.6%). The first two canonical axes were both significant (*p* = 0.001) and together accounted for 36.5% of the total variance (RDA1 = 27.9%, RDA2 = 8.6%). Permutation-based ANOVA identified species as the primary driver of trace element variation (*F* = 10.28, *p* = 0.001, Supplementary Table 2), with red deer aligning with higher Cu concentrations. Cattle showed stronger associations with Co, Mn, Se, and Zn, whereas Fe and Pb contributed more prominently to the profile of horses (Figure 1).

Apart from species effects, several animal and environmental variables contributed significantly to the multivariate structure of trace element profiles. Year of sampling (*F* = 5.35, *p* = 0.001), season (*F* = 6.51, *p* = 0.001), average sward height (*F* = 7.30, *p* = 0.001), and age group (*F* = 3.27, *p* = 0.006) all showed significant effects, indicating that temporal trends, seasonal variation, vegetation structure, and life stage jointly influenced trace element composition. In contrast, sex/pregnancy status and health status did not significantly affect trace element profiles (*p* > 0.1), while body condition showed only a borderline effect (*p* = 0.050), suggesting their limited influence on trace element concentrations (Supplementary Table 2).

#### 3.2.2 Species and element specific associations (regression)

Multivariable linear regression models were constructed for each element separately within each species to quantify associations suggested by the multivariate analysis. Detailed results are shown in Supplementary Tables 3–5. Model fit ranged from weak (adjusted *R*^2^ < 0.10) to strong (up to 0.78 in red deer for Fe), depending on species and element.

**Figure 2:**
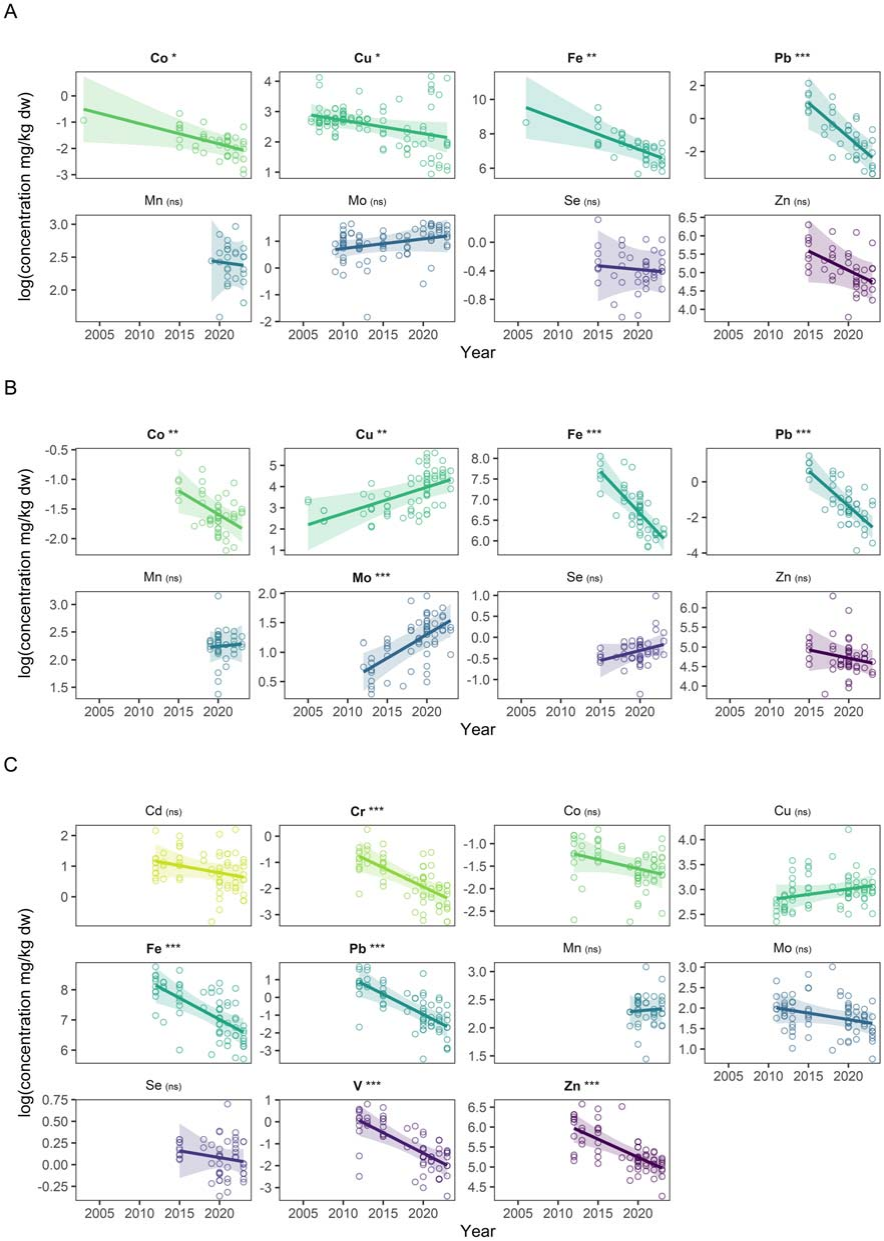
Adjusted effects of year on mineral concentrations in liver across species. **(A)** Heck cattle, **(B)** red deer, and **(C)** Konik horses. Each panel shows the species-specific multivariable regression results for the relationship between year and log-transformed mineral concentrations, adjusted for covariates. Points represent partial residuals; colored lines indicate modeled effects of year with 95% confidence ribbons. Asterisks in facet labels denote significance of the year effect (*p < 0.05; **p < 0.01; ***p < 0.001); (ns) indicates a non-significant trend. Full regression model outputs are available in Supplementary Tables 3–5.

Year of sampling emerged as a consistent predictor across species. Concentrations of Fe and Pb declined significantly over time in all three species (all *p* < 0.01), suggesting a broad temporal trend potentially reflecting environmental changes. Additional year-related declines were observed for concentrations of Co, Cu, and Zn in cattle (*p* < 0.05), as well as Cr, V, and Zn in horses (*p* < 0.001), whereas Mo increased over time in red deer (*p* < 0.001). These temporal patterns are illustrated in Figure 2 and detailed in Supplementary Tables 3–5. Seasonal effects were strongest in red deer and horses, particularly for Fe, Pb, and Se (overall *p* < 0.05). For example, Fe levels increased during spring-summer in red deer (*p* < 0.001) and horses (*p* = 0.032; Supplementary Tables 4, 5). In contrast, season was not a strong factor in cattle models (Supplementary Table 3).

Age related effects were also prominent and often consistent with age-dependent accumulation. In cattle, Cu was significantly lower in senior animals (*p* = 0.046), while Fe, Pb, and Se were lower in younger animals (all *p* < 0.05; Supplementary Table 3). In red deer, young individuals showed lower Fe and Se, but higher Mn and Mo (*p* < 0.02; Supplementary Table 4). The effect of age was more marked in horses, where seniors showed higher concentrations of Cd, Fe, and V, while younger horses had lower levels of these elements (*p* < 0.05; Supplementary Table 5), consistent with possible age-related accumulation. Sex/pregnancy status, body condition, and health status showed generally weak and species-specific associations, with effects mainly evident in red deer, particularly for Co and Pb, which were elevated in pregnant females (*p* = 0.006 and 0.016, respectively) (Supplementary Table 4). These factors were largely not significant predictors in cattle or horses. Overall, these variables explained comparatively little variation in trace element concentrations relative to temporal, seasonal, and age-related factors.

Vegetation structure, represented by annual average sward height of the grasslands and used as a proxy for standing crop and forage availability, was significantly associated with Fe and Pb concentrations in both red deer and horses (all *p* < 0.01; Supplementary Tables 4, 5), while it did not emerge as a significant predictor in cattle models.

#### 3.2.3 Supporting correlation patterns

Spearman rank correlation analyses revealed a range of weak to strong correlations between trace element concentrations in liver within each species (Supplementary Figures 3–5). Across all animal species, the strongest positive correlation was observed between Pb and Fe (ρ > 0.7–0.8), and in horses also between Pb and V (ρ = 0.74). The elements Pb, Co, and Zn frequently showed significant positive correlations with other trace elements, particularly with Fe, Mn, Cr, and Cd, with these patterns being most pronounced in horses (Supplementary Figure 5). Negative correlations were less common, but when present, typically involved Cu, Mo, or Fe, with the strongest inverse relationships detected between Cu and Fe (ρ = −0.54) and Cu and Pb (ρ = −0.49) in red deer (Supplementary Figure 4). These correlation patterns were consistent with the multivariate structure observed in the RDA biplot.

### 3.3 Baseline reference intervals of liver trace element concentrations

**Figure 3:**
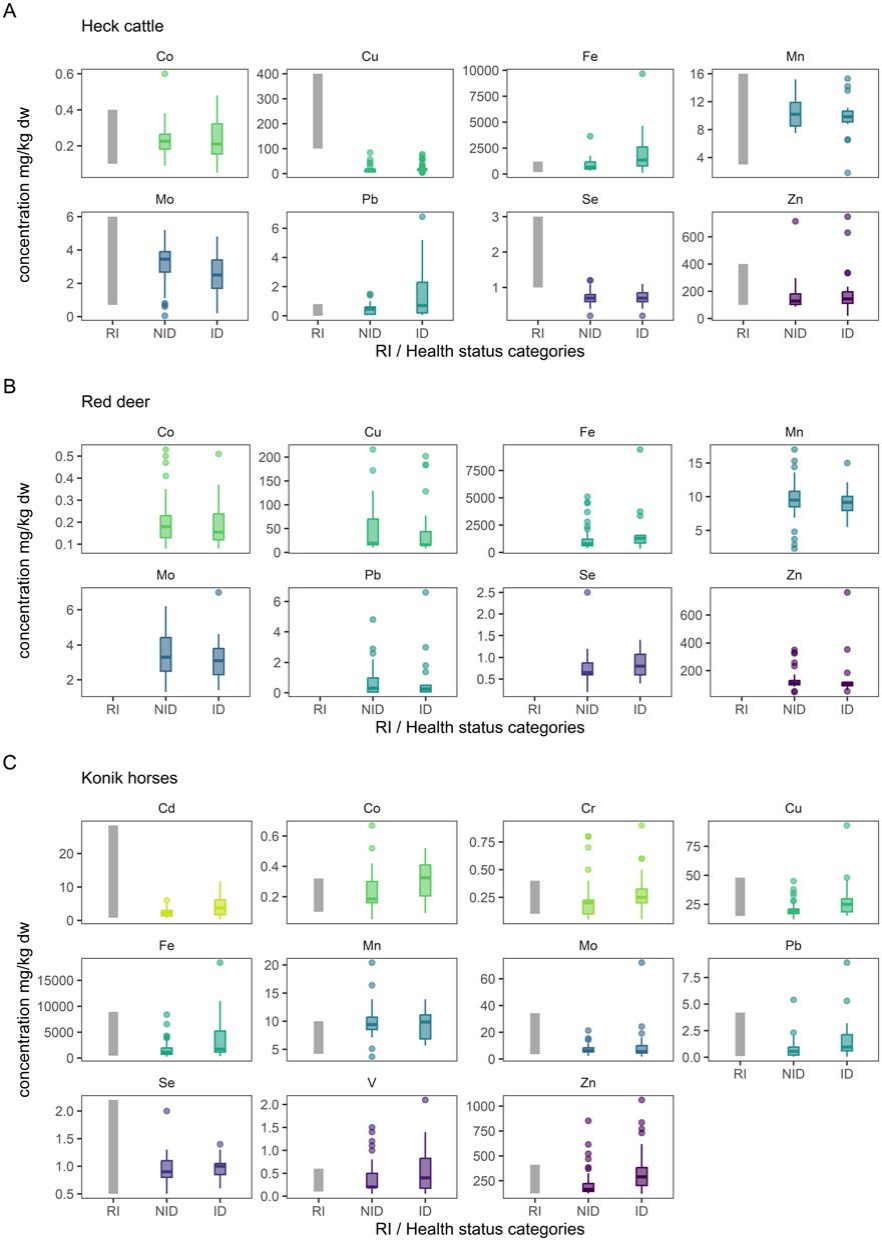
Comparison of reference intervals in livestock and mineral concentrations in the animal populations at the Oostvaardersplassen. **(A)** Heck cattle, **(B)** red deer, and **(C)** Konik horses. Labels on x axis denote: RI, Reference interval livestock; NID, Not indicative of disease; ID, indicative of disease. Reference intervals for cattle and horses are based on Dutch livestock standards (28) and horse study (29). RIs for red deer are not included as formal standard values appear to not be available for this species.

RIs for liver trace element concentrations were estimated separately for Heck cattle (Table 3), red deer (Table 4), and Konik horses (Table 5) from animals classified as “non-indicative of disease.” Measurements of As, Cd, Cr, Ni, and V in both cattle and red deer were more than 50% below the LOQ, while in horses only As and Ni were consistently below the LOQ. For most elements, RIs were wide. In horses, Fe ranged from 314 to nearly 8,000 mg/kg dw, while in cattle and red deer, upper reference limits exceeded 2,500 and 5,000 mg/kg dw, respectively. Overall, the RI analysis confirmed large variation in hepatic trace element concentrations, particularly for Cu, Fe, and Zn, with species-specific ranges. These findings, based on modest sample sizes and appropriate statistical adjustments, emphasize the need for species-specific RIs in health assessments of free-living large herbivores. This can be observed in Figure 3, which shows a visual comparison between established livestock RIs for cattle in the Netherlands (28), as well as, in horses (29) and the trace element concentrations observed for both “non-indicative of disease” and “indicative of disease” groups in the animal populations of OVP. It is apparent that particularly for Cu and Se in Heck cattle compared to livestock the concentrations observed in the area are lower than livestock RIs. This is not observed in horses. Although studies exist on liver trace element concentrations in red deer, established standard RIs for this species are, to the authors' knowledge, not formally determined. Therefore, these are not included, but a side-by-side comparison of both groups can be observed (Figure 3).

**Table 3:** Summary statistics and reference intervals (RIs) for liver mineral concentrations (mg/kg dw) of the subset of Heck cattle at the Oostvaardersplassen included in the “non-indicative of disease” group.

**Element**	***n***	**Mean**	**Sd**	**Median**	**Min**.	**Max**.	**Dist**.	**Method**	95% reference intervals		90% confidence intervals	
									**LRL** ^a^	**URL** ^b^	**LRL**	**URL**
As	22	<0.40	0.32	<0.40	<0.40	1.60	NA^c^	NA	NA	NA	NA	NA
Cd	22	0.15	0.11	0.10	<0.10	0.50	NA	NA	NA	NA	NA	NA
Cr	22	0.16	0.14	0.10	<0.10	0.60	NA	NA	NA	NA	NA	NA
Co	22	0.23	0.11	0.22	<0.10	0.60	NG^d^	P^f^ (T)^g^	<0.10	0.48	0.07–0.12	0.38–0.62
Cu	38	16.47	16.11	16.0	3.0	84.0	NG	R^h^ (T)	1.99	70.50	1.41–3.23	47.21–96.27
Fe	22	950.73	747.89	652.50	327.0	3,644.0	NG	P (T)	232.85	2,578.17	161.2–336.3	1,785.1–3,723.6
Pb	22	0.47	0.43	0.45	<0.10	1.50	NG	R (T)	<0.10	4.57	0.01–0.07	2.26–7.74
Mn	20	10.34	2.17	10.20	7.5	15.2	G^e^	P	6.09	14.58	4.73–7.45	13.22–15.94
Mo	32	3.05	1.35	3.45	<0.10	5.20	NG	R	0.63	6.44	ND^i^	5.60–6.81
Ni	22	0.13	0.12	0.10	<0.10	0.50	NA	NA	NA	NA	NA	NA
Se	22	0.72	0.25	0.70	<0.40	1.20	G	P	<0.40	1.22	0.08–0.38	1.07–1.37
V	22	<0.1	0.03	<0.10	<0.10	0.20	NA	NA	NA	NA	NA	NA
Zn	22	172.41	134.76	128.0	87.0	714.0	NG	R (T)	43.09	383.67	28.51–65.62	222.67–602.97

^a^LRL, Lower reference limit; ^b^URL, Upper reference limit; ^c^NA, not available; ^d^NG, Non-Gaussian; ^e^G, Gaussian; ^f^P, Parametric; ^g^T, Transformed; ^h^R, Robust; ^i^ND, Not determined.

**Table 4:** Summary statistics and reference intervals (RIs) for liver mineral concentrations (mg/kg dw) of the subset of red deer at the Oostvaardersplassen included in the “non-indicative of disease” group.

**Element**	***n***	**Mean**	**Sd**	**Median**	**Min**.	**Max**.	**Dist**.	**Method**	95% reference intervals		90% confidence intervals	
									**LRL** ^a^	**URL** ^b^	**LRL**	**URL**
As	42	<0.40	0.07	<0.40	<0.40	0.50	NA^c^	NA	NA	NA	NA	NA
Cd	42	0.13	0.10	0.10	<0.10	0.40	NA	NA	NA	NA	NA	NA
Cr	42	0.50	1.03	0.10	<0.10	3.90	NA	NA	NA	NA	NA	NA
Co	42	0.20	0.11	0.18	<0.10	0.53	NG^d^	NP^f^	<0.10	0.53	0.08–0.09	0.46–0.53
Cu	57	45.82	44.14	19.0	10.0	216.0	NG	NP	10.45	196.20	10.0–10.90	110.0–216.0
Fe	42	1,278.2	1,202.7	789.5	370.0	5,097.0	NG	NP	370.68	5,057.93	370.0–419.73	4,363.9–5,097.0
Pb	42	0.68	0.98	0.30	<0.10	4.80	NG	NP	<0.10	4.66	ND^h^	2.53–4.80
Mn	33	9.52	3.25	9.50	2.30	17.0	G^e^	P^g^	3.15	15.88	1.57–4.74	14.29–17.46
Mo	57	3.44	1.19	3.30	1.30	6.20	G	P	1.10	5.78	0.66–1.55	5.34–6.23
Ni	42	0.21	0.48	<0.10	<0.10	2.0	NA	NA	NA	NA	NA	NA
Se	42	0.74	0.34	0.65	<0.40	2.50	NG	NP	<0.40	2.40	0.20–0.50	1.10–2.50
V	42	<0.10	0.10	<0.10	<0.10	0.60	NA	NA	NA	NA	NA	NA
Zn	42	130.93	69.05	109.50	45.0	350.0	NG	NP	45.38	348.65	45.0–83.23	313.53–350.0

^a^LRL, Lower reference limit; ^b^URL, Upper reference limit; ^c^NA, not available; ^d^NG, Non-Gaussian; ^e^G, Gaussian; ^f^NP, Non-parametric; ^g^P, Parametric; ^h^ND, Not determined.

**Table 5:** Summary statistics and reference intervals (RIs) for liver mineral concentrations (mg/kg dw) of the subset of Konik horses at the Oostvaardersplassen included in the “non-indicative of disease” group.

**Element**	***n***	**Mean**	**Sd**	**Median**	**Min**.	**Max**.	**Dist**.	**Method**	95% reference intervals		90% confidence intervals	
									**LRL** ^a^	**URL** ^b^	**LRL**	**URL**
As	48	<0.40	0.0	<0.40	<0.40	<0.40	NA^c^	NA	NA	NA	NA	NA
Cd	48	2.43	1.30	2.25	0.60	6.0	NG^d^	NP^e^	0.65	5.89	0.60–0.85	4.89–6.0
Cr	48	0.21	0.18	0.20	<0.10	0.80	NG	NP	<0.10	0.80	ND^g^	0.61–0.80
Co	48	0.23	0.12	0.18	<0.10	0.67	NG	NP	<0.10	0.64	0.05–0.09	0.40–0.67
Cu	53	20.23	6.06	19.0	12.0	45.0	NG	NP	12.70	42.55	12.0–15.0	32.55–45.0
Fe	48	1,726.4	1,599.1	1,129.0	274.0	8,383.0	NG	NP	314.05	7,968.8	274.0–481.18	4,058.1–8,383.0
Pb	48	0.76	0.90	0.55	<0.10	5.40	NG	NP	<0.10	4.70	0.05–0.06	1.90–5.40
Mn	34	9.86	3.06	9.40	3.70	20.40	NG	R^f^	2.98	15.64	1.0–5.20	13.30–17.70
Mo	53	7.17	3.22	6.30	2.40	21.30	NG	NP	2.68	19.27	2.40–3.73	12.80–21.30
Ni	48	0.19	0.46	<0.10	<0.10	2.90	NA	NA	NA	NA	NA	NA
Se	38	0.97	0.28	0.90	0.50	2.0	NG	R	<0.40	1.53	0.19–0.55	1.34–1.69
V	48	0.39	0.36	0.20	<0.10	1.50	NG	NP	<0.10	1.48	ND	1.11–1.50
Zn	48	218.71	141.49	164.0	123.0	852.0	NG	NP	123.2	798.7	123.0–127.0	476.9–852.0

^a^LRL, Lower reference limit; ^b^URL, Upper reference limit; ^c^NA, not available; ^d^NG, Non-Gaussian; ^e^NP, Non-parametric; ^f^R, Robust; ^g^ND, Not determined.

Sensitivity analyses were performed for trace elements in which statistical extremes were identified, while no outliers were detected for the remaining elements. In cattle, exclusion of statistical outliers resulted in modest changes RI estimates for Cu and Mo, with upper RI limits changing by less than 5% and overall RI widths decreasing by less than 10%. For Co and Se in cattle, exclusion of statistical outliers reduced the sample size below the minimum threshold recommended for RI estimation (*n* < 20), and RIs could therefore not be calculated in the sensitivity analysis. In red deer and horses, larger effects of outlier exclusion were observed for selected trace elements, particularly Mn, Se, Zn, Mo, and Cu. In these cases, exclusion of statistical outliers generally resulted in higher lower RI limits, lower upper RI limits, and substantial reductions in overall RI width (approximately 20%−70%), accompanied by narrowing of CI widths. For many other trace elements across all species, no statistical outliers were identified and RI estimates were unchanged (Supplementary Tables 6–8).

## 4 Discussion

To evaluate liver trace element concentrations in free-living large herbivores at the OVP nature reserve in the Netherlands, this study established species-specific RIs for 13 elements in the livers of Heck cattle, Konik horses, and red deer. The panel of elements included essential nutrients (Cr, Co, Cu, Fe, Mn, Mo, Se, Zn) and non-essential elements of toxicological concern, which are also considered indicative of industrial pollution (As, Cd, Pb, Ni, V). Trace element concentrations were analyzed in relation to species, sampling year, and biological variables, including age and season. The results showed clear interspecies differences and temporal variation in several elements, which are relevant for both health and environmental monitoring. While this study is constrained by sample size and the challenges inherent to wildlife health classification, it provides a first reference point for interpreting liver trace elements in these minimally managed populations. These findings offer a practical foundation for ongoing monitoring in the OVP and could inform similar efforts in other nature reserves with a multi species assemblage of large herbivores.

### 4.1 Animal and environmental determinants of liver trace element concentrations

Trace element concentrations in livers of animals are influenced by factors such as feed, water, environment, and management, and different species tend to have distinct trace element profiles due to their unique biological, physiological, and habitat characteristics (2). In this study, such species-specific differences were clearly reflected in the element profiles of Heck cattle, Konik horses, and red deer at the OVP, as shown by multivariate analysis (RDA), with distinct clustering patterns observed for each species. Although some overlap occurred, differences in feeding and foraging behaviors, as well as physiological and metabolic variations among species, are likely to have contributed to the observed variation in trace element composition (2, 14, 30). Specifically, the separation of species appeared to be driven by higher hepatic Cu concentrations in red deer, and by increased Fe and Pb concentrations in horses. These element distribution patterns may reflect both dietary specialization and species-specific physiological processes that affect key kinetic parameters such as rates of absorption and elimination. It may be hypothesized, for example, that red deer may access relatively copper-rich food sources unavailable or less favored by cattle and horses, or may have inherently higher liver Cu storage levels due to a higher rate of absorption or a lower rate of elimination compared to the other species. Similarly, it may be hypothesized that the higher Fe and Pb in horses might result from their grazing methods leading to increased soil ingestion, or a tendency to bioaccumulate these metals over time. The specific reason(s) for species differences were outside of the scope of this investigation, but distinct patterns within the data may offer insight into possible causes of variation.

Age group related variation was demonstrated in this analysis, particularly in horses, which may be explained by the fact that younger animals typically have higher mineral demands to support growth, while older individuals may show evidence of cumulative exposure over time, resulting in increased levels of some elements in body tissues (2, 29). This pattern is especially relevant for non-essential or potentially toxic elements such as Pb and Cd, which tend to accumulate in tissues over time and with age (2, 31). This is consistent with the significantly higher hepatic Cd concentrations observed in older horses in the present study, as well as higher Pb concentrations in older horses and cattle, although the latter did not reach statistical significance. For essential elements, an age-related increase was also observed for Fe in horses. Fe is subject to physiological regulation, and the observed age-related hepatic increase in horses may reflect progressive storage from long-term exposure and/or metabolic changes with age (2, 32). Further research, such as dietary analyses and environmental sampling, is needed to better understand the relative contributions of diet, behavior, and physiology to mineral trace element profiles in these herbivore species coexisting at the OVP.

Body condition and health status did not consistently contribute to variation in trace element profiles, especially when considering other factors. From a nutritional and physiological perspective, it could be expected that animals with better body condition and/or health status would have more adequate (but not excessive) concentrations of essential trace elements such as Cu, Se, and Zn, and lower concentrations of non-essential and potentially toxic elements such as Pb and Cd, reflecting more optimal nutrition and greater physiological reserves; however, no consistent associations between these elements and body condition or health status were observed in this study. One possible explanation is that adaptation to local conditions may allow animals at the OVP to tolerate wider trace element ranges, compared to the same species in conventionally managed or production settings, without obvious effects on health (6).

Sex and reproductive status can also alter trace element requirements, as pregnant and lactating females have increased demands for specific minerals such as Cu, Se, Zn, and Fe to support fetal development and milk production (2). While reproductive stage differences were most evident in red deer at the OVP, uneven and small subgroup sample sizes likely reduced statistical power, limiting the detection of more nuanced associations. Despite these challenges, the observed consistency across species and statistical methods improves confidence in the general trends described here and provides a base for future, more detailed studies of trace element dynamics in free-living herbivores.

Environmental factors such as year of sampling, season and sward height also contributed to element variation. These patterns likely reflect general ecological influences on trace element exposure, including changes in the mineral content of forage and soil over time, influenced by environmental factors such as temperature and soil type (33). The specific mechanisms are complex and may involve interactions between plant, soil, and animal factors (13, 33). Overall, it should be acknowledged that it is difficult to disentangle all these biological and environmental factors, as not all relevant factors could be measured, and other unaccounted variables may also influence liver element concentrations. In addition, because trace element concentrations were not measured simultaneously in environmental matrices such as soil, vegetation, or water, it is not possible to directly attribute hepatic concentrations to specific environmental sources; environmental interpretations should therefore be considered inferential and require future validation with integrated environmental sampling.

Heavy metal exposure across large herbivores at the OVP was generally low, with most concentrations falling below the LOQ or below concerning levels. This likely reflects the relatively low environmental contamination in the reserve, consistent with previous assessments of water (34). For certain element such as As and Cd, along with Cr, Ni, and V, measured concentrations were consistently below the LOQ, suggesting limited environmental exposure and providing important baseline data from which future environmental trends and pollutant accumulation could potentially be inferred. Although these low concentrations prevent definitive classification, any future detections above LOQ could potentially indicate unusually high exposure for these animal populations. The decreasing trend observed in Pb and Fe concentrations over the study period may reflect long-term environmental changes affecting trace element availability, such as shifts in soil properties, forage composition, or management practices, patterns also reported in cattle and wild herbivores (13, 19, 35). Additionally, in the case of Pb, this trend could also potentially be consistent with broader declines in environmental heavy metal emissions in Europe from anthropogenic sources following regulatory measures (36). However, direct attribution is not possible in the absence of concurrent environmental measurements, and these temporal associations should be interpreted with caution due to uneven sampling across years and potential confounding by population structure, environmental variability, or sampling effort differences. Ni and V are not considered essential for animal health, but if detected in increased concentrations, they may serve as indicators of environmental contamination and be valuable for pollution assessment (30).

### 4.2 Interpretation of baseline reference intervals of liver trace element concentrations

Regarding RIs in cattle at the OVP, these were comparable with cattle from other nature areas in the Netherlands (12). The most notable differences were in Fe, with a higher upper limit of ~2,500 mg/kg dw in the current study compared to 693 mg/kg dw reported in other areas, and a narrower RI for Se (< 0.4–1.22 mg/kg dw in this study and 0.6–5.5 mg/kg dw from a comparable study) (12). The RI for Cu (1.99–70.50 mg/kg dw) was lower than the bovine reference levels established for livestock (6, 28), but similar to values reported in wilderness settings within the Netherlands (12). It should be noted that these national livestock RIs are based primarily on intensively managed dairy cattle and are the available values for comparison, but not the ideal physiological reference for free-living cattle breeds such as Heck cattle. Importantly, lower Cu concentrations in free-living cattle should not be directly interpreted as evidence of deficiency. Unlike production animals, these populations are not selected for traits such as high growth rates, reproductive output, or milk production, and in the case of the OVP, these animals are generally managed without supplementary feeding, with some exceptions since 2018, in which hay was offered if the average body condition score of Heck cattle was below two. It is possible that their physiological Cu requirements are lower, or that they have adapted to a lower range of Cu intake without clinical signs of deficiency. This may explain why animals in both this and previous studies appeared clinically healthy despite liver Cu levels that would be considered marginal or deficient in livestock (6, 12). A similar pattern was observed in Konik horses and red deer at the OVP, where Cu levels were generally below reference levels derived from intensively managed or supplemented populations. However, for all three animal species at the OVP, low Cu levels were comparable with other wild or free-living species, which also had lower Cu levels than the domestic cattle range (35, 37, 38). Cu deficiency can negatively impact ruminant health, and lead to enzootic ataxia in red deer (39). Additionally, it has been reported that low growth rate in red deer was associated with low Cu (40), and a “spectacled” appearance around the eye was observed in bison (38). While at the OVP no specific Cu deficiency clinical signs were observed, differences between livestock ranges and those observed at the OVP do not rule out the possibility of subclinical deficiency in these populations, highlighting the need for caution when applying livestock thresholds to free-living animals and supporting the case for species- and context-specific RIs.

Regarding Se, levels detected in red deer at the OVP were similar to values reported in other studies (35, 37, 41). Both RIs of Heck cattle and red deer at the OVP were overall lower than for domestic cattle (6, 28). Se deficiency is documented in both livestock and farmed wild large herbivores, and severe Se deficiency in herbivores is known to cause diseases such as white muscle disease (2, 39, 42). However, Se deficiency can occur without obvious clinical signs and can lead to subclinical effects that compromise animal health (42). At the OVP, although subclinical or marginal Se status could not be ruled out, animals in the area have historically not shown clear signs of Se deficiency, and all animals included in the RI estimation showed no evident pathological findings.

RIs of horses at the OVP were largely consistent with those reported for slaughtered horses in the Netherlands (29). The only notable difference was the upper limit for Zn, which was ~800 mg/kg dw in this study, about twice the upper limit observed for slaughtered horses (29). This variation could reflect differences in environmental exposure or forage composition at the OVP, although further research is needed to fully capture the reasons behind this difference. In contrast, the RIs for Fe were remarkably similar between the horses of the OVP and the slaughtered horses populations, with both studies reporting high upper limits near 8,000 mg/kg dw (29), which is above the considered toxic range (6). This similarity is particularly interesting given that the horses in the reported study were mostly privately owned and managed, whereas the OVP horses are free-ranging, unmanaged, and before 2018 subsisted entirely on natural forage and water sources. Previous research has suggested a possible link between elevated hepatic Fe in Dutch horses and the high iron concentrations in surface waters (29). Internal water quality assessments from the OVP indicate that natural water sources often contain elevated concentrations of mineral elements, including Fe, and are generally considered, compared to standards for livestock, unsuitable for consumption (34).

Despite this, horses included in RI estimation showed no evident disease or iron-associated pathology on routine gross and histopathological examination. However, these diagnostic approaches cannot exclude subclinical iron accumulation or early iron-associated hepatopathy, as specific iron histochemical staining (e.g., Perls' Prussian blue) was not applied systematically across the reference population. Iron storage disease includes both hemosiderosis (non-pathological accumulation) and hemochromatosis (pathological deposition with tissue damage), documented in horses, although rarely (31). Elevated hepatic Fe concentrations exceeding commonly cited toxic thresholds have also been documented in apparently healthy Dutch horses, potentially reflecting subclinical effects from chronic exposure undetectable by routine macroscopic examination (29). Thus, while OVP horses show age-related Fe accumulation consistent with long-term exposure, subclinical iron-associated hepatopathy remains a possibility requiring targeted histochemistry for confirmation. The same trend of elevated upper RI limits for Fe was also observed in Heck cattle and red deer in the current study, suggesting a possible ecosystem-level exposure. Comparing Fe concentrations in liver across other unmanaged herbivore populations in different environments may help determine whether these high values are exceptional or typical of specific habitat types. More broadly, free-living and wild animals, such as roe deer and wild boar, as well as domestic cattle, have been shown to act as sentinels for environmental trace element exposure in natural habitats (30, 43). Additionally, such data may provide contextual information relevant for livestock management in nature-inclusive farming systems, although direct comparisons require caution.

To the authors' knowledge, formal standard RIs specifically established for red deer are not available, unlike for cattle and horses. However, available data detail trace element concentrations in various deer species (e.g., red deer, roe deer, reindeer) and other large herbivores in natural areas, such as European bison (35, 37, 38, 44, 45), which can serve as useful comparative references. While some studies measure trace element elements in liver, others utilize hair samples, with the latter increasingly favored for their non-invasive collection method, which is particularly important in wildlife research (46). Nonetheless, comparisons between different sample types require caution, as some studies report positive correlations between trace element concentrations in hair and internal organs, while others report negative correlations (5). Although comparisons with other deer species or populations from different habitats can provide useful context, ecological, dietary, and physiological differences may limit the relevance of direct reference comparisons. Notably, published studies report geographic variation in trace element concentrations within cervid populations, underlining the need for broader cross-species and cross-habitat research to clarify the extent to which specific RIs can be generalized or require local calibration to account for environmental and physiological variability (35, 45, 47).

In general, RIs are also established for smaller subgroups, such as different age classes and sexes (3, 4). While age appeared to influence trace element concentrations in the OVP populations, subgroup-specific RI partitioning was not possible due to insufficient sample sizes. Uniform sampling across age and sex categories should be prioritized in future monitoring to allow for this level of analysis. Establishing reference intervals ideally requires a large number of healthy individuals, which supports the use of non-parametric methods and improves the reliability of confidence intervals (3, 4, 48). A minimum of 120 reference individuals is recommended, as smaller sample sizes increase uncertainty in the estimation of limits (4, 48). However, in wildlife studies, obtaining such large numbers of well-defined healthy individuals is often challenging due to difficulties in health assessment and limited sample availability, as shown by evaluations of laboratory reference data from non-domestic species (7).

While ASVCP recommends ≥120 individuals, some reference group sizes in this study were smaller. Although parametric and robust methods were applied in accordance with ASVCP guidelines, limited sample sizes resulted in relatively wide confidence intervals, which may affect the precision of the estimated limits. In this context, the values presented here should be regarded as baseline reference values for free-living herbivore populations at the OVP that can be refined as additional data become available. In the present study, this limitation was also addressed by classifying animals based on post-mortem pathology reports, allowing for a relatively accurate classification between healthy and diseased groups. Although this approach does not completely eliminate uncertainty, it provides a biologically meaningful compromise given the limitations of working with non-farmed free-living animals. It should also be acknowledged that the absence of gross or histological lesions does not guarantee that an animal was in an optimal physiological or nutritional state, and subclinical nutritional stress may still influence hepatic trace elements despite normal pathology. Although about 70% of animals in the reference group had good or normal body condition, a subset with moderate body condition was retained when no pathological abnormalities were present. Excluding these animals would have further reduced sample sizes and increased uncertainty in RI estimation, illustrating a trade-off between biological rigour and statistical feasibility in wildlife studies.

Although most sampled Heck cattle were classified as “indicative of disease,” this likely reflects the conservative health classification criteria applied rather than poor general health in the population. Many cattle showed hepatic lesions or adult parasites consistent with liver fluke (*Fasciola hepatica*) infection, in line with the high seroprevalence previously reported at the OVP (15). Because trace elements were measured in liver, animals with relevant hepatic pathology were excluded from the reference population to avoid potential confounding effects, which strengthens biological validity but reduces sample size, particularly in cattle. These constraints highlight the need for alternative approaches in wildlife RI estimation, such as indirect methods that leverage larger mixed datasets using statistical modeling (49, 50). However, applying these methods in non-farmed free-living or wild animal populations remains challenging due to sparse and heterogeneous data, a difficulty reflected in the variable quality and completeness of published wildlife laboratory reference data (7). At the OVP, continued sampling and monitoring with an increased sample size and more balanced representation across and within years, as well as age groups, will be essential to refine future RI estimates and improve their precision and reliability.

Some outlier values were observed in the dataset. However, after thorough verification to exclude processing errors, these values were retained in all analyses due to the health classification criteria applied during animal selection for RIs estimation. While this approach ensures that only biologically reasonable data from apparently healthy individuals were included, it also means that there was the potential of some genuine physiological variation or undetected subclinical conditions that could influence the RIs. Outlier identification in RI studies is inherently dependent on statistical definitions and distributional assumptions, and in heterogeneous, free-ranging wildlife populations, extreme values may reflect genuine biological variability rather than analytical artifacts. The sensitivity analysis done in this study showed that RI estimates for cattle were relatively robust to outlier exclusion, whereas for some elements in red deer and horses, exclusion resulted in narrower RI and CI widths and shifts in upper and lower limits. In a few cases, outlier exclusion reduced sample size below the minimum recommended for RI estimation, highlighting the sensitivity of RI estimates in small datasets. These findings indicate that while retaining outliers preserves potential biological variability, RI estimates are best interpreted as baseline ranges rather than fixed diagnostic thresholds. Future studies with larger sample sizes and more detailed health assessments could help clarify the impact of such outliers on RI robustness.

## 5 Conclusion

In conclusion, the findings clearly show that element profiles vary substantially between Heck cattle, Konik horses, and red deer, and are correlated with multiple biological and environmental factors, including age, season, and year of sampling, with differences observed between RIs in these populations and their farmed counterparts. These results highlight the limitations of using livestock-derived RIs as a reference for health assessments in free-living minimally managed animals, as the physiological, ecological, and environmental conditions in which these animals live differ from those of conventionally managed livestock populations.

Establishing species-specific RIs generally offers a more appropriate, and possibly more reliable basis for monitoring the health status of large herbivores in nature reserves and contributes to more accurate interpretation of trace element concentrations in post-mortem surveillance. Further research is needed to assess whether the RIs determined in the current study can be applied to similar species in other nature reserves, or if location-specific values are required. Comparing trace element concentrations across different habitats, in vegetation and soil, would help determine whether these reference intervals are broadly applicable or specific to an ecosystem. While more comparative research is needed, this study could serve as an example for similar studies and longitudinal trace element monitoring in nature reserves across the Netherlands and Europe. Improving trace element monitoring in free-living animals will strengthen health surveillance in nature areas and provide better tools for evaluating both animal health and long-term environmental trends. This could be beneficial for areas with multiple species, where identifying trace element imbalances could inform management strategies and decision-making.

## Data Availability

The datasets presented in this article are not readily available because they belong to Staatsbosbeheer and restrictions apply to the availability of these data. Data can be made available upon reasonable request and with permission of Staatsbosbeheer. Requests to access the datasets should be directed to Perry Cornelissen, p.cornelissen@staatsbosbeheer.nl.
