# Supplementary material for: Monitoring trace minerals and heavy metals in liver of free-living large herbivores in the Netherlands
Source: Front Vet Sci. 2026 Feb 24;13:1751586. doi: 10.3389/fvets.2026.1751586 (PMC12971517; doi:10.3389/fvets.2026.1751586)
Supplement: Supplementary file 1 [file Data_Sheet_1.pdf]

Supplementary Material

Monitoring trace minerals and heavy metals in liver of free-living large herbivores in the Netherlands

Inês Marcelino*, Gustavo Monti, Perry Cornelissen, Evelyn Bassingthwaighte, Jasper het Lam, Deon Van der Merwe, Wim H.M. Van der Poel

***Correspondence:** Inês Marcelino, ines.marcelino@wur.nl

1 Health status classification

The health status of animals sampled was classified using the flow chart below (Supplementary Figure 1).

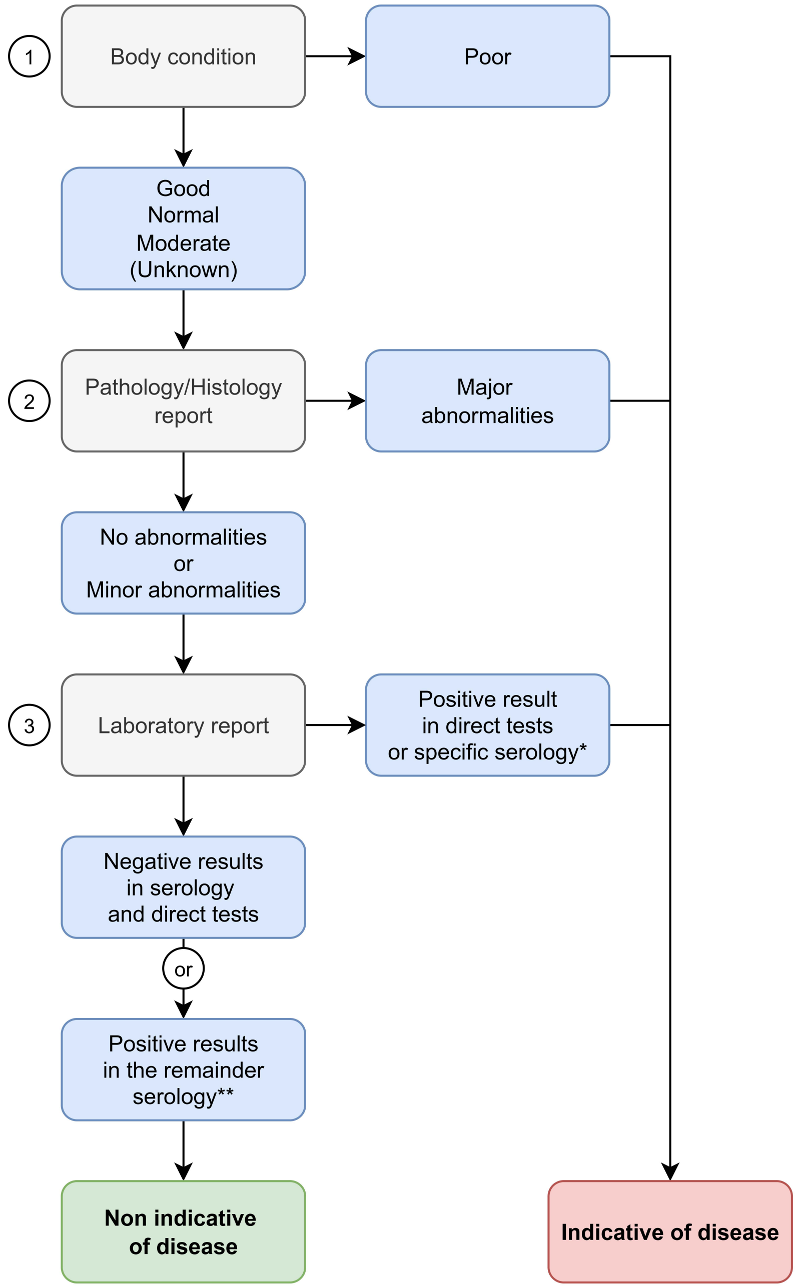

Supplementary Figure 1 Health status flowchart used to assign animals into two distinct postmortem health status categories: Indicative of disease and Non indicative of disease. *Positive results for MAP, Salmonella spp., bTB, B. abortus, EHV-1 and 4, BVDV antigen. **Positive results for BoHV-1, BVDV antibody, BLV, BTV, SBV, EIAV, EIV, FMDV, WNV, C. burnetii, L. Hardjo, S. Dublin, S. Typhimurium, and F. hepatica.

Classification of health status was first based on the external condition of the animal, as recorded in the pathology report (step 1 in the flowchart). Animals culled due to clear signs of apparent disease or with poor body condition were directly classified as Indicative of disease. Animals with good to moderate body condition underwent further assessment based on pathology/histology report (step 2). If major abnormalities were identified, the classification was also Indicative of disease. If no abnormalities or only minor findings were observed, the classification then depended on laboratory results (step 3). Positive results in direct tests (*MAP*, *Salmonella* spp., bTb, *B. abortus*, EHV-1 and 4) or specific serology (BVDV antigen) were considered confirmatory, and the animal was classified as Indicative of disease. Finally, if all results were negative or if for some specific serology the result was positive (BoHV-1, BVDV antibody, BLV, BTV, SBV, EIAV, EIV, FMDV, WNV, *C. burnetii*, *L.* Hardjo, *S.* Dublin, *S.* Typhimurium, and *F. hepatica*) then the animals were classified as Non indicative of disease, since it was assumed that a positive serology result for the listed pathogens indicates past infection and not active infection. It is important to note that mineral element concentrations (either designated low or high in the reports) were not considered in the classification, since the goal was to evaluate mineral concentrations. Additionally, because these were animals living freely, grazing all year round with no anti-parasitic treatment, presence of gastrointestinal parasites was considered usual. Any traumatic lesions caused by euthanasia/culling were not considered abnormalities.

Abnormalities were classified as:

- Major: animal was culled due to external signs of disease; chronic and/or extensive findings/lesions in the pathology report; findings/lesions that clearly indicate disease process; high burden of gastrointestinal parasites.
- Minor: findings/lesions classified as minor and/or localised in pathology report; unspecific findings/lesions that do not clearly indicate disease process; moderate burden of gastrointestinal parasites.
- No abnormalities: no findings/lesions described in pathology report; minor burden of gastrointestinal parasites.

Supplementary Table 1 Body condition of 'non indicative of disease' classified animals.

Body condition*, n (%)	Heck cattle	Red deer	Konik horses
**Good**	22 (57.9)	30 (52.6)	21 (39.6)
**Normal**	7 (18.4)	15 (26.3)	14 (26.4)
**Moderate**	4 (10.5)	10 (17.5)	16 (30.2)
**Unknown**	5 (13.2)	2 (3.5)	2 (3.8)
**Total**	38	57	53

*From the Dutch: *goed* (good), *normaal* (normal), *matig* (moderate).

2 Reference interval estimation methods

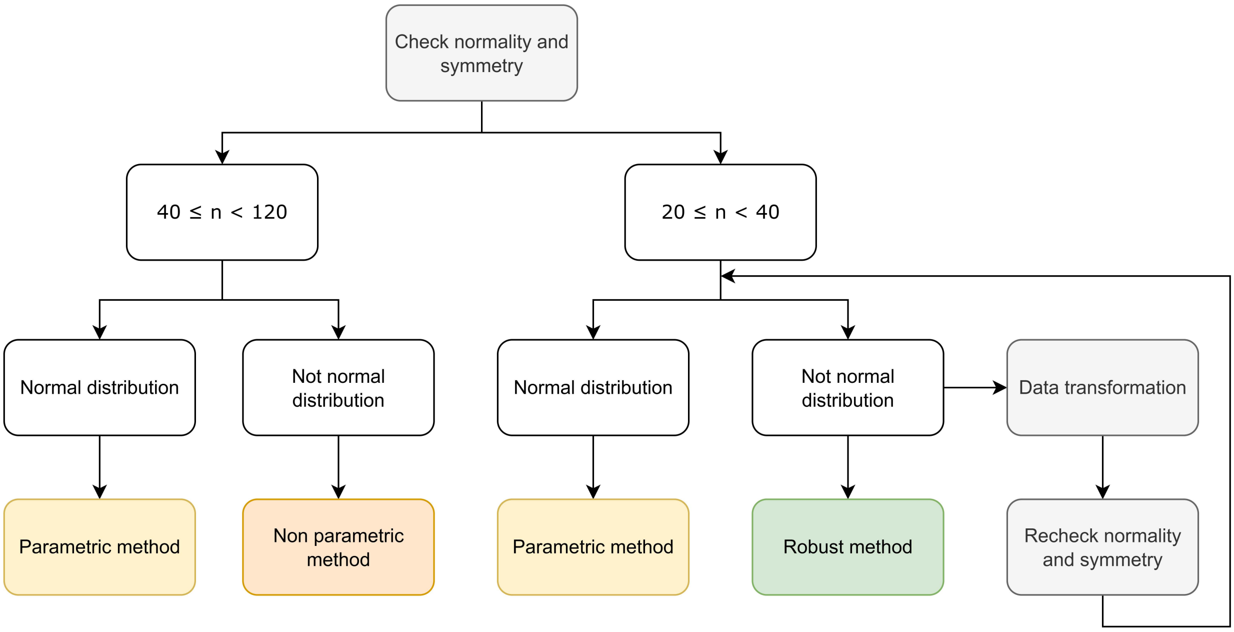

Supplementary Figure 2 Flowchart illustrating the decision process for selecting the appropriate statistical method to calculate reference intervals for liver mineral concentrations in the animal populations at the *Oostvaardersplassen*, based on sample size, data distribution and symmetry.

3 Redundancy analysis (RDA)

Supplementary Table 2 Permutation-based ANOVA results from Redundancy Analysis (RDA). Marginal tests were used to assess the independent effect of each predictor on mineral concentrations, with 999 permutations. Adjusted % of total variance of the overall model was 36.6%. Significant predictors ($p < 0.05$) are highlighted in bold.

Test	df	Variance	F	*p*-value	% of Total Variance
**Overall model**	13	1.693	6.456	**0.001**	43.3
**Canonical axes**					
RDA1	1	1.090	47.19	**0.001**	27.9
RDA2	1	0.338	14.63	**0.001**	8.6
**Predictors**					
Species	2	0.490	10.28	**0.001**	—
Year	1	0.128	5.347	**0.001**	—
Age group	2	0.156	3.272	**0.006**	—
Sex/Pregnancy	2	0.074	1.561	0.137	—
Body condition	1	0.066	2.788	0.050	—
Health status	1	0.028	1.175	0.310	—
Season	1	0.155	6.508	**0.001**	—
Avg. plant height per year	1	0.174	7.296	**0.001**	
Residuals	93	2.218	—	—	—

Group sizes for categorical predictors were as follows: Species – cattle (n = 26), red deer (n = 38), horses (n = 41); Age group – young (n = 15), adult (n = 81), senior (n = 9); Sex/Pregnancy – pregnant female (n = 25), non-pregnant female (n = 29), male (n = 51); Body condition – not poor (n = 100), poor (n = 5); Health status – no indication of disease (n = 77), indication of disease (n = 28); Season – autumn/winter (n = 65), spring/summer (n = 40).

4 Regression analysis

Supplementary Table 3 Regression models for element concentrations in Heck cattle liver at the *Oostvaardersplassen*. Model type was selected per element based on residual diagnostics. All models are linear models with log-transformed outcomes, except for Mo and Se (raw scale). Shown are coefficients, 95% confidence intervals, *p*-values, and adjusted R^2. Predictors include year, age group (ref: adult), sex/pregnancy status (ref: non-pregnant female), body condition (ref: not poor), health status (ref: diseased), season (ref: autumn–winter), and average vegetation height.

Element	n	Variable	Coef.	95% CI	*p*-value	Adjusted R^2
**Co**	43	Year	-0.078	-0.145 – -0.011	**0.025**	0.134
		Age group – senior	0.015	-0.452 – 0.481	0.950	
		Age group – young	-0.468	-0.952 – 0.016	0.058	
		Sex/Pregnancy – pregnant female	-0.072	-0.568 – 0.425	0.771	
		Sex/Pregnancy – male	0.268	-0.154 – 0.691	0.206	
		Body condition – poor	0.249	-0.357 – 0.856	0.409	
		Health status – not diseased	0.299	-0.095 – 0.692	0.132	
		Season – spring-summer	0.102	-0.307 – 0.511	0.615	
		Avg. Plant height per year	-0.013	-0.098 – 0.072	0.763	
**Cu**	119	Year	-0.044	-0.085 – -0.002	**0.040**	0.132
		Age group – senior	-0.354	-0.702 – -0.006	**0.046**	
		Age group – young	-0.134	-0.417 – 0.148	0.349	
		Sex/Pregnancy – pregnant female	-0.116	-0.419 – 0.187	0.450	
		Sex/Pregnancy – male	0.125	-0.196 – 0.445	0.443	
		Body condition – poor	0.021	-0.259 – 0.300	0.883	
		Health status – not diseased	-0.038	-0.379 – 0.302	0.823	
		Season – spring-summer	-0.292	-0.638 – 0.054	0.097	
		Avg. Plant height per year	-0.028	-0.099 – 0.044	0.446	
**Fe**	43	Year	-0.173	-0.286 – -0.059	**0.004**	0.381
		Age group – senior	0.261	-0.451 – 0.973	0.461	
		Age group – young	-0.832	-1.569 – -0.095	**0.028**	
		Sex/Pregnancy – pregnant female	-0.109	-0.839 – 0.620	0.762	
		Sex/Pregnancy – male	-0.357	-1.003 – 0.288	0.268	
		Body condition – poor	0.094	-0.765 – 0.953	0.825	
		Health status – not diseased	0.223	-0.397 – 0.843	0.469	
		Season – spring-summer	-0.249	-0.912 – 0.415	0.451	
		Avg. Plant height per year	0.000	-0.125 – 0.124	0.994	
**Pb**	42	Year	-0.415	-0.636 – -0.194	**0.001**	0.492
		Age group – senior	0.319	-0.860 – 1.498	0.585	
		Age group – young	-1.457	-2.530 – -0.383	**0.009**	
		Sex/Pregnancy – pregnant female	0.760	-0.368 – 1.888	0.179	
		Sex/Pregnancy – male	0.205	-0.735 – 1.146	0.660	
		Body condition – poor	0.296	-1.108 – 1.700	0.670	
		Health status – not diseased	0.614	-0.301 – 1.528	0.181	
		Season – spring-summer	-0.258	-1.224 – 0.709	0.590	
		Avg. Plant height per year	0.003	-0.183 – 0.189	0.974	

**Mn**	26	Year	-0.017	-0.197 – 0.163	0.842	0.069
		Age group – senior	0.143	-0.844 – 1.130	0.763	
		Age group – young	-0.264	-0.844 – 0.316	0.349	
		Sex/Pregnancy – pregnant female	-0.233	-0.886 – 0.419	0.459	
		Sex/Pregnancy – male	0.171	-0.231 – 0.573	0.381	
		Body condition – poor	0.586	-0.736 – 1.908	0.362	
		Health status – not diseased	0.205	-0.224 – 0.633	0.326	
		Season – spring-summer	0.210	-0.298 – 0.718	0.394	
		Avg. Plant height per year	0.034	-0.106 – 0.174	0.615	
**Mo**	79	Year	0.076	-0.008 – 0.160	0.076	0.353
		Age group – senior	0.001	-0.606 – 0.608	0.998	
		Age group – young	-0.505	-1.062 – 0.051	0.074	
		Sex/Pregnancy – pregnant female	0.113	-0.446 – 0.671	0.688	
		Sex/Pregnancy – male	0.612	-0.052 – 1.276	0.070	
		Body condition – poor	-0.147	-0.694 – 0.400	0.595	
		Health status – not diseased	0.085	-0.469 – 0.638	0.761	
		Season – spring-summer	1.039	0.442 – 1.636	**0.001**	
		Avg. Plant height per year	0.121	-0.004 – 0.245	0.058	
**Se**	42	Year	-0.006	-0.047 – 0.034	0.756	0.197
		Age group – senior	-0.049	-0.265 – 0.167	0.644	
		Age group – young	-0.319	-0.515 – -0.122	**0.002**	
		Sex/Pregnancy – pregnant female	-0.099	-0.306 – 0.107	0.335	
		Sex/Pregnancy – male	0.062	-0.110 – 0.234	0.469	
		Body condition – poor	-0.049	-0.306 – 0.208	0.700	
		Health status – not diseased	0.053	-0.115 – 0.220	0.525	
		Season – spring-summer	-0.022	-0.199 – 0.155	0.805	
		Avg. Plant height per year	-0.037	-0.071 – -0.003	**0.035**	
**Zn**	42	Year	-0.105	-0.223 – 0.012	0.078	0.170
		Age group – senior	-0.345	-0.973 – 0.284	0.272	
		Age group – young	-0.179	-0.751 – 0.393	0.528	
		Sex/Pregnancy – pregnant female	-0.483	-1.084 – 0.118	0.111	
		Sex/Pregnancy – male	0.487	-0.014 – 0.988	0.057	
		Body condition – poor	0.580	-0.168 – 1.328	0.124	
		Health status – not diseased	-0.064	-0.551 – 0.424	0.792	
		Season – spring-summer	0.074	-0.441 – 0.589	0.772	
		Avg. Plant height per year	0.035	-0.065 – 0.134	0.483	

Supplementary Table 4 Regression models for element concentrations in red deer liver at the *Oostvaardersplassen*. Model type was selected per element based on residual diagnostics. All models are linear models with log-transformed outcomes. Shown are coefficients, 95% confidence intervals, *p*-values, and adjusted R^2. Predictors include year, age group (ref: adult), sex/pregnancy status (ref: non-pregnant female), body condition (ref: not poor), health status (ref: diseased), season (ref: autumn–winter), and average vegetation height.

Element	n	Variable	Coef.	95% CI	p-value	Adjusted R^2
Co	51	Year	-0.079	-0.139 – -0.020	0.001	0.598
		Age group – senior	-0.185	-0.554 – 0.183	0.316	
		Age group – young	0.155	-0.089 – 0.400	0.207	
		Sex/Pregnancy – pregnant female	0.377	0.114 – 0.640	**0.006**	
		Sex/Pregnancy – male	-0.188	-0.448 – 0.072	0.152	
		Body condition – poor	0.804	0.052 – 1.555	**0.037**	
		Health status – not diseased	0.336	0.111 – 0.561	**0.004**	
		Season – spring-summer	0.362	0.111 – 0.612	**0.006**	
		Avg. Plant height per year	0.044	0.002 – 0.085	**0.040**	
**Cu**	70	Year	0.118	0.038 – 0.198	**0.005**	0.155
		Age group – senior	-0.778	-1.701 – 0.146	0.097	
		Age group – young	-0.303	-0.803 – 0.197	0.231	
		Sex/Pregnancy – pregnant female	0.170	-0.445 – 0.785	0.582	
		Sex/Pregnancy – male	-0.053	-0.560 – 0.454	0.836	
		Body condition – poor	0.823	-0.177 – 1.824	0.105	
		Health status – not diseased	-0.026	-0.597 – 0.544	0.927	
		Season – spring-summer	-0.324	-0.792 – 0.145	0.172	
		Avg. Plant height per year	-0.057	-0.154 – 0.039	0.240	
**Fe**	51	Year	-0.204	-0.275 – -0.133	**<0.001**	0.777
		Age group – senior	0.155	-0.288 – 0.598	0.483	
		Age group – young	-0.431	-0.725 – -0.137	**0.005**	
		Sex/Pregnancy – pregnant female	0.106	-0.210 – 0.423	0.500	
		Sex/Pregnancy – male	0.225	-0.087 – 0.537	0.154	
		Body condition – poor	0.868	-0.035 – 1.771	0.059	
		Health status – not diseased	0.018	-0.253 – 0.289	0.894	
		Season – spring-summer	0.792	0.491 – 1.093	**<0.001**	
		Avg. Plant height per year	0.155	0.105 – 0.204	**<0.001**	
**Pb**	51	Year	-0.393	-0.556 – -0.230	**<0.001**	0.675
		Age group – senior	0.299	-0.716 – 1.314	0.555	
		Age group – young	0.088	-0.585 – 0.761	0.793	
		Sex/Pregnancy – pregnant female	0.904	0.179 – 1.628	**0.016**	
		Sex/Pregnancy – male	0.008	-0.707 – 0.724	0.981	
		Body condition – poor	1.722	-0.348 – 3.791	0.101	
		Health status – not diseased	0.326	-0.294 – 0.946	0.294	
		Season – spring-summer	1.082	0.392 – 1.772	**0.003**	
		Avg. Plant height per year	0.229	0.115 – 0.343	**0.0002**	
**Mn**	38	Year	0.014	-0.087 – 0.116	0.775	0.345
		Age group – senior	-0.049	-0.491 – 0.393	0.821	

		Age group – young	0.380	0.067 – 0.692	**0.019**	
		Sex/Pregnancy – pregnant female	0.012	-0.306 – 0.330	0.939	
		Sex/Pregnancy – male	-0.544	-0.852 – -0.236	**0.001**	
		Health status – not diseased	0.082	-0.202 – 0.366	0.560	
		Season – spring-summer	0.020	-0.319 – 0.360	0.904	
		Avg. Plant height per year	-0.013	-0.087 – 0.061	0.720	
**Mo**	62	Year	0.079	0.039 – 0.120	**0.0002**	0.322
		Age group – senior	-0.208	-0.588 – 0.171	0.276	
		Age group – young	0.292	0.058 – 0.525	**0.015**	
		Sex/Pregnancy – pregnant female	0.147	-0.115 – 0.409	0.265	
		Sex/Pregnancy – male	-0.084	-0.313 – 0.146	0.469	
		Body condition – poor	0.050	-0.395 – 0.496	0.821	
		Health status – not diseased	0.132	-0.103 – 0.367	0.266	
		Season – spring-summer	0.184	-0.009 – 0.378	0.062	
		Avg. Plant height per year	0.004	-0.038 – 0.047	0.842	
**Se**	51	Year	0.048	-0.016 – 0.111	0.140	0.210
		Age group – senior	-0.014	-0.412 – 0.384	0.945	
		Age group – young	-0.270	-0.534 – -0.006	**0.045**	
		Sex/Pregnancy – pregnant female	0.068	-0.216 – 0.352	0.632	
		Sex/Pregnancy – male	-0.211	-0.492 – 0.069	0.136	
		Body condition – poor	0.826	0.014 – 1.637	**0.046**	
		Health status – not diseased	-0.001	-0.244 – 0.242	0.992	
		Season – spring-summer	0.314	0.043 – 0.585	**0.024**	
		Avg. Plant height per year	-0.009	-0.054 – 0.035	0.676	
**Zn**	51	Year	-0.042	-0.128 – 0.044	0.325	0.168
		Age group – senior	-0.247	-0.783 – 0.289	0.357	
		Age group – young	-0.060	-0.416 – 0.295	0.734	
		Sex/Pregnancy – pregnant female	0.062	-0.320 – 0.445	0.744	
		Sex/Pregnancy – male	0.041	-0.337 – 0.419	0.829	
		Body condition – poor	1.170	0.077 – 2.264	**0.037**	
		Health status – not diseased	0.187	-0.141 – 0.514	0.256	
		Season – spring-summer	0.344	-0.020 – 0.709	0.063	
		Avg. Plant height per year	0.060	0.000 – 0.120	**0.050**	

Supplementary Table 5 Regression models for element concentrations in Konik horses liver at the Oostvaardersplassen. Model type was selected per element based on residual diagnostics. All models are linear models with log-transformed outcomes, except for Co and V (raw scale). Shown are coefficients, 95% confidence intervals, *p*-values, and adjusted R^2. Predictors include year, age group (ref: adult), sex/pregnancy status (ref: non-pregnant female), body condition (ref: not poor), health status (ref: diseased), season (ref: autumn–winter), and average vegetation height.

Element	n	Variable	Coef.	95% CI	p-value	Adjusted R^2
**Cd**	68	Year	-0.047	-0.122 – 0.028	0.211	0.304
		Age group – senior	0.416	0.002 – 0.830	**0.049**	
		Age group – young	-0.592	-1.050 – -0.134	**0.012**	
		Sex/Pregnancy – pregnant female	0.179	-0.328 – 0.686	0.483	
		Sex/Pregnancy – male	-0.123	-0.497 – 0.250	0.511	
		Body condition – poor	0.142	-0.390 – 0.675	0.595	
		Health status – not diseased	-0.163	-0.615 – 0.289	0.474	
		Season – spring-summer	0.422	0.053 – 0.792	**0.026**	
		Avg. Plant height per year	0.010	-0.082 – 0.101	0.836	
**Cr**	68	Year	-0.146	-0.224 – -0.067	**0.0005**	0.393
		Age group – senior	-0.038	-0.471 – 0.396	0.863	
		Age group – young	-0.331	-0.810 – 0.149	0.173	
		Sex/Pregnancy – pregnant female	-0.213	-0.744 – 0.317	0.424	
		Sex/Pregnancy – male	-0.226	-0.617 – 0.164	0.251	
		Body condition – poor	0.399	-0.158 – 0.956	0.157	
		Health status – not diseased	0.061	-0.412 – 0.535	0.796	
		Season – spring-summer	0.485	0.099 – 0.871	**0.015**	
		Avg. Plant height per year	0.073	-0.023 – 0.169	0.131	
**Co**	68	Year	-0.011	-0.024 – 0.002	0.084	0.371
		Age group – senior	-0.008	-0.078 – 0.062	0.820	
		Age group – young	0.100	0.022 – 0.178	**0.013**	
		Sex/Pregnancy – pregnant female	0.017	-0.069 – 0.103	0.693	
		Sex/Pregnancy – male	0.038	-0.026 – 0.101	0.236	
		Body condition – poor	0.106	0.016 – 0.197	**0.023**	
		Health status – not diseased	0.018	-0.059 – 0.095	0.637	
		Season – spring-summer	0.051	-0.012 – 0.114	0.110	
		Avg. Plant height per year	0.000	-0.016 – 0.015	0.952	
**Cu**	79	Year	0.022	-0.013 – 0.057	0.212	0.078
		Age group – senior	0.008	-0.202 – 0.217	0.942	
		Age group – young	0.161	-0.045 – 0.366	0.123	
		Sex/Pregnancy – pregnant female	-0.070	-0.324 – 0.184	0.584	
		Sex/Pregnancy – male	-0.032	-0.214 – 0.150	0.728	
		Body condition – poor	0.171	-0.079 – 0.421	0.177	
		Health status – not diseased	-0.127	-0.336 – 0.082	0.231	
		Season – spring-summer	0.000	-0.177 – 0.178	0.999	
		Avg. Plant height per year	-0.028	-0.074 – 0.019	0.243	
**Fe**	68	Year	-0.142	-0.220 – -0.064	**0.0006**	0.524
		Age group – senior	0.819	0.390 – 1.249	**0.0003**	

		Age group – young	-0.556	-1.032 – -0.080	**0.023**	
		Sex/Pregnancy – pregnant female	0.129	-0.397 – 0.656	0.625	
		Sex/Pregnancy – male	0.070	-0.318 – 0.457	0.720	
		Body condition – poor	0.472	-0.080 – 1.025	0.093	
		Health status – not diseased	-0.044	-0.514 – 0.425	0.850	
		Season – spring-summer	0.421	0.037 – 0.804	**0.032**	
		Avg. Plant height per year	0.112	0.017 – 0.207	**0.022**	
**Pb**	68	Year	-0.228	-0.339 – -0.117	**0.0001**	0.427
		Age group – senior	0.345	-0.270 – 0.961	0.266	
		Age group – young	-0.100	-0.782 – 0.581	0.770	
		Sex/Pregnancy – pregnant female	-0.087	-0.841 – 0.667	0.818	
		Sex/Pregnancy – male	-0.214	-0.769 – 0.340	0.442	
		Body condition – poor	0.491	-0.300 – 1.283	0.219	
		Health status – not diseased	-0.082	-0.755 – 0.590	0.808	
		Season – spring-summer	0.910	0.361 – 1.458	**0.002**	
		Avg. Plant height per year	0.205	0.069 – 0.341	**0.004**	
**Mn**	41	Year	0.012	-0.092 – 0.117	0.814	-0.111
		Age group – senior	-0.048	-0.466 – 0.369	0.815	
		Age group – young	0.086	-0.419 – 0.590	0.731	
		Sex/Pregnancy – pregnant female	0.135	-0.288 – 0.558	0.519	
		Sex/Pregnancy – male	0.255	-0.059 – 0.569	0.107	
		Body condition – poor	-0.086	-0.558 – 0.387	0.714	
		Health status – not diseased	0.015	-0.313 – 0.342	0.928	
		Season – spring-summer	-0.068	-0.318 – 0.182	0.582	
		Avg. Plant height per year	-0.041	-0.103 – 0.021	0.190	
**Mo**	79	Year	-0.031	-0.076 – 0.014	0.171	0.358
		Age group – senior	0.438	0.165 – 0.711	**0.002**	
		Age group – young	-0.068	-0.336 – 0.200	0.613	
		Sex/Pregnancy – pregnant female	0.343	0.012 – 0.673	**0.043**	
		Sex/Pregnancy – male	-0.108	-0.346 – 0.129	0.366	
		Body condition – poor	0.304	-0.022 – 0.631	0.067	
		Health status – not diseased	0.066	-0.206 – 0.339	0.629	
		Season – spring-summer	0.186	-0.046 – 0.417	0.114	
		Avg. Plant height per year	0.117	0.056 – 0.178	**0.0003**	
**Se**	53	Year	-0.016	-0.065 – 0.034	0.529	0.073
		Age group – senior	-0.179	-0.404 – 0.046	0.116	
		Age group – young	-0.170	-0.418 – 0.078	0.175	
		Sex/Pregnancy – pregnant female	0.147	-0.100 – 0.393	0.238	
		Sex/Pregnancy – male	0.010	-0.162 – 0.181	0.909	
		Body condition – poor	0.107	-0.130 – 0.344	0.367	
		Health status – not diseased	0.120	-0.068 – 0.307	0.206	
		Season – spring-summer	0.187	0.023 – 0.352	**0.027**	
		Avg. Plant height per year	-0.002	-0.038 – 0.035	0.928	
**V**	68	Year	-0.090	-0.129 – -0.052	**<0.001**	0.468
		Age group – senior	0.222	0.009 – 0.434	**0.041**	
		Age group – young	-0.234	-0.469 – 0.001	0.051	

		Sex/Pregnancy – pregnant female	0.082	-0.178 – 0.342	0.531	
		Sex/Pregnancy – male	-0.038	-0.230 – 0.153	0.689	
		Body condition – poor	0.172	-0.101 – 0.445	0.213	
		Health status – not diseased	0.062	-0.170 – 0.294	0.596	
		Season – spring-summer	0.155	-0.034 – 0.344	0.107	
		Avg. Plant height per year	0.059	0.012 – 0.105	**0.015**	
**Zn**	68	Year	-0.091	-0.138 – -0.043	**0.0004**	0.528
		Age group – senior	-0.079	-0.343 – 0.185	0.552	
		Age group – young	0.359	0.066 – 0.651	**0.017**	
		Sex/Pregnancy – pregnant female	-0.025	-0.348 – 0.298	0.878	
		Sex/Pregnancy – male	0.169	-0.069 – 0.407	0.160	
		Body condition – poor	0.479	0.139 – 0.818	**0.007**	
		Health status – not diseased	0.060	-0.229 – 0.348	0.681	
		Season – spring-summer	0.121	-0.114 – 0.357	0.306	
		Avg. Plant height per year	0.022	-0.036 – 0.081	0.446	

5 Correlation analysis

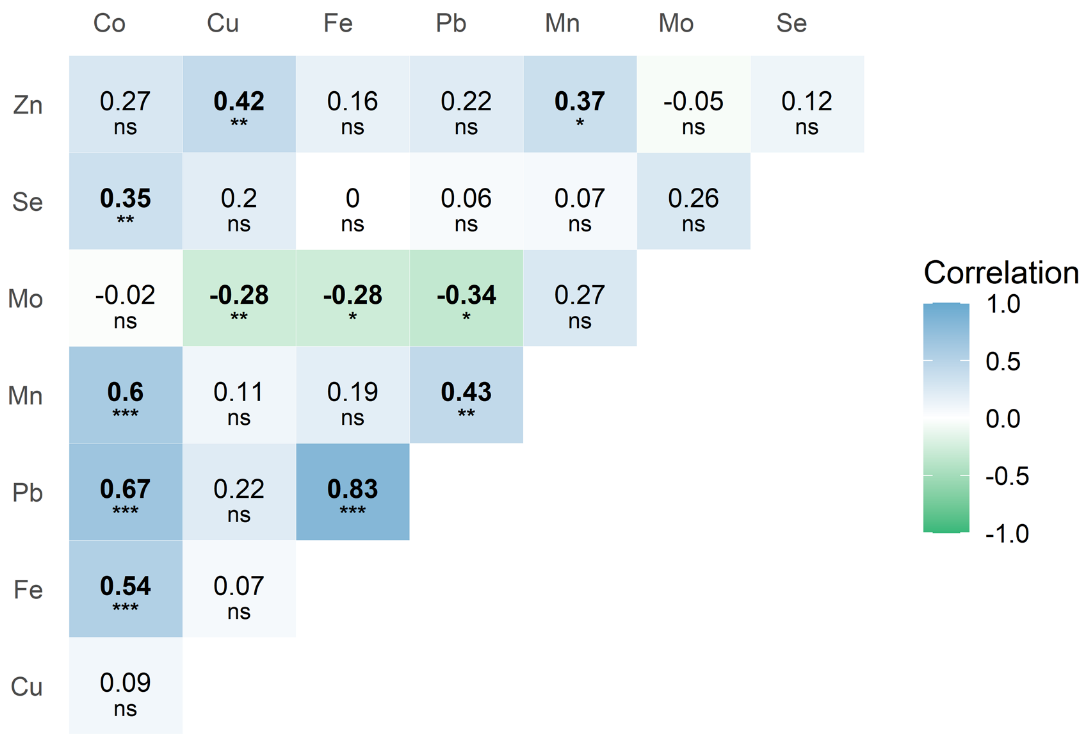

Supplementary Figure 3 Spearman rank correlation coefficients between elements in the liver of Heck cattle at the Oostvaardersplassen. The colour of the cells represents the strength and direction of the correlation, with blue indicating positive correlations and green indicating negative correlations, as shown in the colour scale. Only the upper triangle of the matrix is shown. Statistical significance is denoted as follows: $p \leq 0.001$ (***), $p \leq 0.01$ (**), $p \leq 0.05$ (*), and *ns* indicates non-significant correlations ($p > 0.05$).

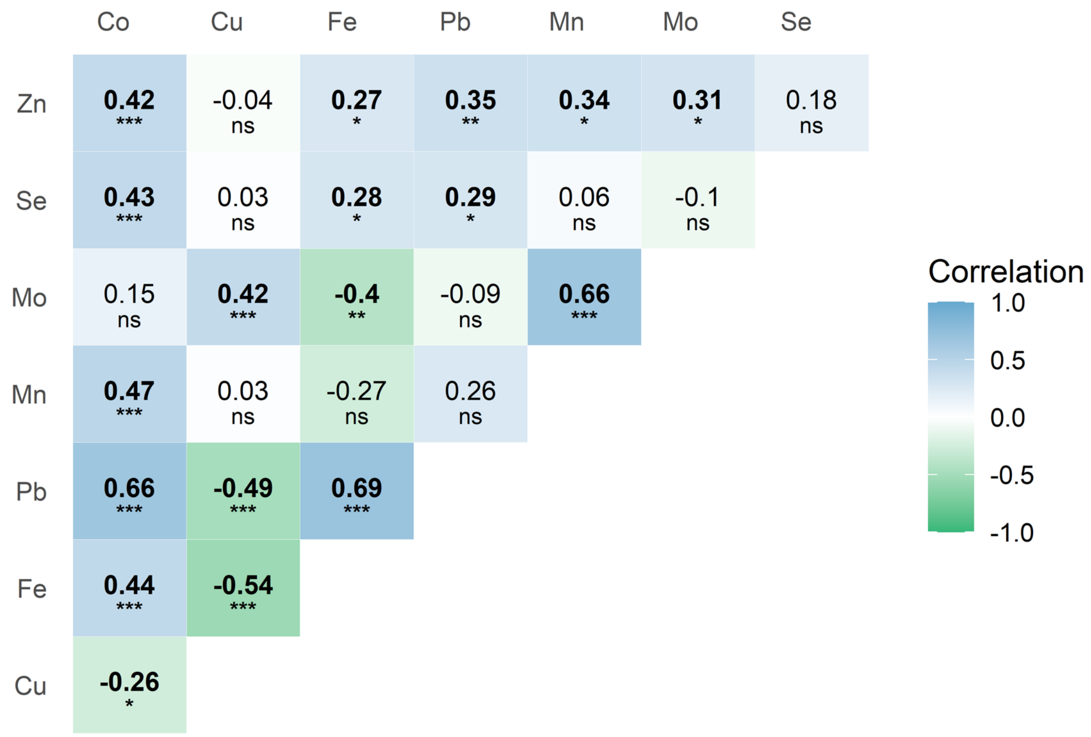

Supplementary Figure 4 Spearman rank correlation coefficients between elements in the liver of red deer at the Oostvaardersplassen. The colour of the cells represents the strength and direction of the correlation, with blue indicating positive correlations and green indicating negative correlations, as shown in the colour scale. Only the upper triangle of the matrix is shown. Statistical significance is denoted as follows: $p \leq 0.001$ (***), $p \leq 0.01$ (**), $p \leq 0.05$ (*), and *ns* indicates non-significant correlations ($p > 0.05$).

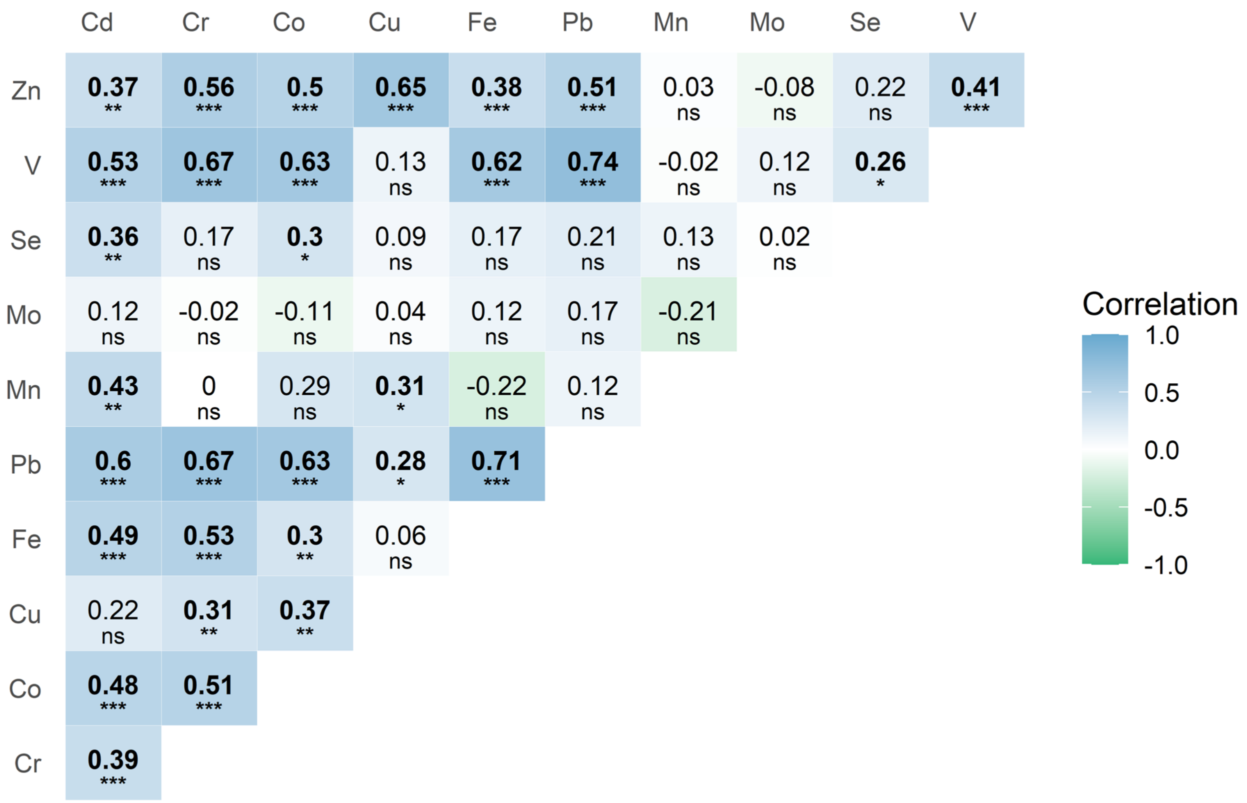

Supplementary Figure 5 Spearman rank correlation coefficients between elements in the liver of Konik horses at the Oostvaardersplassen. The colour of the cells represents the strength and direction of the correlation, with blue indicating positive correlations and green indicating negative correlations, as shown in the colour scale. Only the upper triangle of the matrix is shown. Statistical significance is denoted as follows: $p \leq 0.001$ (***), $p \leq 0.01$ (**), $p \leq 0.05$ (*), and *ns* indicates non-significant correlations ($p > 0.05$).

6 Sensitivity analysis for mineral reference limits

For sensitivity analyses, percent change in the lower (LRL) and upper (URL) reference limits was calculated as the relative difference between sensitivity (statistical outliers removed) and primary estimates, expressed as a percentage. Reference interval (RI) width was defined as the difference between the upper and lower reference limits. Percent change in RI width was calculated as the relative difference between sensitivity and primary RI widths, expressed as a percentage. Confidence interval (CI) width was defined as the difference between the upper and lower bounds of the 90% CI for each reference limit. Percent changes in CI width were calculated separately for the lower and upper reference limits and expressed relative to the primary analysis. Negative values indicate a decrease and positive values an increase relative to the primary RI estimates. When exclusion of statistical outliers reduced sample size below the minimum recommended threshold for RI estimation (n < 20), or when CIs could not be reliably estimated due to statistical limitations (e.g., limited data variability), results were classified as not determined (ND). When no statistical outliers were identified, sensitivity analysis was not applicable (NA).

Supplementary Table 6 Sensitivity analysis of reference interval (RI) estimates for liver mineral concentrations in Heck cattle at the Oostvaardersplassen.

Element	*n* primary	Outliers removed	*n* sensitivity	Primary RI	Sensitivity RI	% change in LRL	% change in URL	% change in RI width	% change in CI for LRL	% change in CI for URL
**Co**	22	3	19	<0.10 – 0.48	ND (n < 20)	ND	ND	ND	ND	ND
**Cu**	38	2	36	1.99 – 70.50	2.29 – 67.06	15.08	-4.88	-5.46	6.04	-6.52
**Fe**	22	0	22	232.85 – 2578.17	NA	NA	NA	NA	NA	NA
**Pb**	22	0	22	<0.10 – 4.57	NA	NA	NA	NA	NA	NA
**Mn**	20	0	20	6.09 – 14.58	NA	NA	NA	NA	NA	NA
**Mo**	32	1	31	0.63 – 6.44	0.85 – 6.22	33.78	-3.32	-7.36	ND	-5.85
**Se**	22	3	19	<0.40 – 1.22	ND (n < 20)	ND	ND	ND	ND	ND
**Zn**	22	0	22	43.09 – 383.67	NA	NA	NA	NA	NA	NA

Supplementary Table 7 Sensitivity analysis of reference interval (RI) estimates for liver mineral concentrations in red deer at the Oostvaardersplassen.

**Element**	***n* primary**	**Outliers removed**	***n* sensitivity**	**Primary RI**	**Sensitivity RI**	**% change in LRL**	**% change in URL**	**% change in RI width**	**% change in CI for LRL**	**% change in CI for URL**
**Co**	42	0	42	<0.10 – 0.53	NA	NA	NA	NA	NA	NA
**Cu**	57	0	57	10.45 – 196.20	NA	NA	NA	NA	NA	NA
**Fe**	42	0	42	370.68 – 5057.93	NA	NA	NA	NA	NA	NA
**Pb**	42	0	42	<0.10 – 4.66	NA	NA	NA	NA	NA	NA
**Mn**	33	7	26	3.15 – 15.88	6.47 – 13.04	104.99	-17.85	-48.31	-41.77	-41.77
**Mo**	57	0	57	1.10 – 5.78	NA	NA	NA	NA	NA	NA
**Se**	42	2	40	<0.40 – 2.40	0.50 – 1.20	124.72	-50.16	-68.00	ND	-92.50
**Zn**	42	7	35	45.38 – 348.65	83.0 – 172.0	82.92	-50.67	-70.65	-92.15	-56.13

Supplementary Table 8 Sensitivity analysis of reference interval (RI) estimates for liver mineral concentrations in Konik horses at the Oostvaardersplassen.

Element	*n* primary	Outliers removed	*n* sensitivity	Primary RI	Sensitivity RI	% change in LRL	% change in URL	% change in RI width	% change in CI for LRL	% change in CI for URL
**Cd**	48	0	48	0.65 – 5.89	NA	NA	NA	NA	NA	NA
**Cr**	48	0	48	<0.10 – 0.80	NA	NA	NA	NA	NA	NA
**Co**	48	1	47	0.06 – 0.636	0.08 – 0.64	44.49	0.59	-3.71	-42.86	-2.99
**Cu**	53	3	50	12.70 – 42.55	14.28 – 34.45	12.40	-19.04	-32.41	-66.67	-39.36
**Fe**	48	0	48	314.05 – 7968.8	NA	NA	NA	NA	NA	NA
**Pb**	48	0	48	<0.10 – 4.70	NA	NA	NA	NA	NA	NA
**Mn**	34	4	30	2.98 – 15.64	5.47 – 12.97	83.76	-17.08	-40.78	-53.42	-45.37
**Mo**	53	2	51	2.68 – 19.27	3.23 – 15.14	20.52	-21.43	-28.21	-32.08	-50.62
**Se**	38	2	36	<0.4 – 1.53	0.50 – 1.41	40.94	-8.08	-22.82	-51.84	-42.02
**V**	48	5	43	0.05 – 1.48	0.10 – 1.49	100.0	0.85	-2.63	ND	-12.82
**Zn**	48	0	48	123.2 – 798.7	NA	NA	NA	NA	NA	NA
